# Sex differences in the association between diabetes and cancer: a systematic review and meta-analysis of 121 cohorts including 20 million individuals and one million events

**DOI:** 10.1007/s00125-018-4664-5

**Published:** 2018-07-20

**Authors:** Toshiaki Ohkuma, Sanne A. E. Peters, Mark Woodward

**Affiliations:** 1 The George Institute for Global Health, University of New South Wales, Level 10, King George V Building, Royal Prince Alfred Hospital, Missenden Rd, Camperdown, NSW 2050 Australia; 2 The George Institute for Global Health, University of Oxford, Le Gros Clark Building, South Parks Road, Oxford, OX1 3QX UK; 3 Department of Epidemiology, Johns Hopkins University, Baltimore, MD USA

**Keywords:** Cancer, Diabetes, Meta-analysis, Sex differences, Systematic review

## Abstract

**Aims/hypothesis:**

Diabetes has been shown to be a risk factor for some cancers. Whether diabetes confers the same excess risk of cancer, overall and by site, in women and men is unknown.

**Methods:**

A systematic search was performed in PubMed for cohort studies published up to December 2016. Selected studies reported sex-specific relative risk (RR) estimates for the association between diabetes and cancer adjusted at least for age in both sexes. Random-effects meta-analyses with inverse-variance weighting were used to obtain pooled sex-specific RRs and women-to-men ratios of RRs (RRRs) for all-site and site-specific cancers.

**Results:**

Data on all-site cancer events (incident or fatal only) were available from 121 cohorts (19,239,302 individuals; 1,082,592 events). The pooled adjusted RR for all-site cancer associated with diabetes was 1.27 (95% CI 1.21, 1.32) in women and 1.19 (1.13, 1.25) in men. Women with diabetes had ~6% greater risk compared with men with diabetes (the pooled RRR was 1.06, 95% CI 1.03, 1.09). Corresponding pooled RRRs were 1.10 (1.07, 1.13) for all-site cancer incidence and 1.03 (0.99, 1.06) for all-site cancer mortality. Diabetes also conferred a significantly greater RR in women than men for oral, stomach and kidney cancer, and for leukaemia, but a lower RR for liver cancer.

**Conclusions/interpretation:**

Diabetes is a risk factor for all-site cancer for both women and men, but the excess risk of cancer associated with diabetes is slightly greater for women than men. The direction and magnitude of sex differences varies by location of the cancer.

**Electronic supplementary material:**

The online version of this article (10.1007/s00125-018-4664-5) contains peer-reviewed but unedited supplementary material, which is available to authorised users.

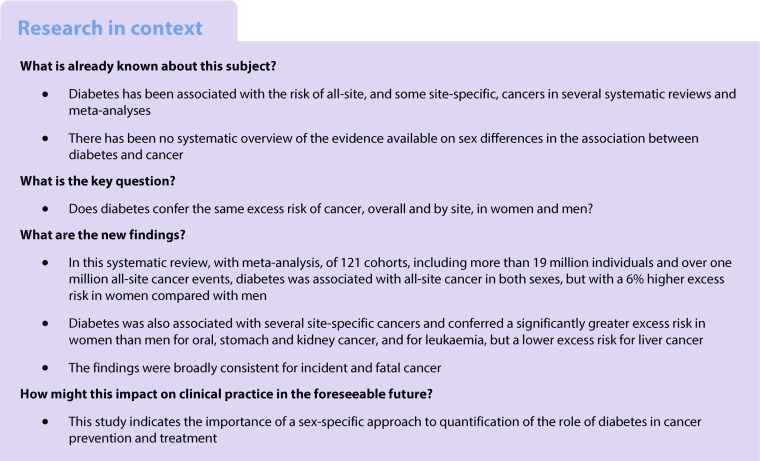



## Introduction

Cancer is the second leading causes of death in the world [1]. In 2015, there were 17.5 million incident cancer cases and 8.7 million cancer deaths globally, and it is estimated that one in four women and one in three men develop cancer during their lifetime [2]. The incidence of cancer is expected to increase in the next decades, emphasising the importance of efficient prevention and treatment of cancer worldwide.

The prevalence of diabetes has also grown rapidly. In 2015, one in 11 adults (415 million) were reported to have diabetes, five million deaths were attributed to diabetes, and 12% of global health expenditure was spent on diabetes and its complications [3]. Diabetes has been associated with the risk of all-site and some site-specific cancers in several systematic reviews and meta-analyses [4–13]. However, only a minority of these associations are based on robust supporting evidence without question of significant bias [14]. To date, there has been no systematic overview of the evidence available on sex differences in the association between diabetes and cancer. We have previously published compelling evidence that women with diabetes are at an increased risk of stroke [15], coronary heart disease [16] and dementia [17] compared with their male peers. We now question whether this is also true for cancer. In this study, we conducted the most comprehensive systematic review and meta-analysis, to date, to estimate the relative effect of diabetes on the risk of cancer in women compared with men.

## Methods

### Search strategy and selection criteria

A systematic search was performed in PubMed (https://www.ncbi.nlm.nih.gov/pubmed/) on 23 December 2016 using a combined text word and medical subject heading search strategy (electronic supplementary material [ESM] Table 1). The reference lists of identified reports were also checked for other potentially relevant studies.

Observational cohort studies in general populations were included if they had provided relative risks (RRs), or equivalents, for the association between diabetes and cancer in both women and men. Studies were excluded if they had not adjusted at least for age or did not provide information about the variability around the point estimate, or if they only had data for one sex. In case of duplicate reports from the same study, the study providing the longest follow-up or the highest number of cases was included. We also used individual participant data from the Asia Pacific Cohort Studies Collaboration (APCSC) [18], treated as two separate combinations of data from cohorts in Asia and cohorts from Australia or New Zealand, as in our previous work [15, 16]. One author (TO) did the search and extracted the data. Uncertainties regarding the inclusion or exclusion of articles and data extraction were discussed by all authors and resolved by mutual consent. The meta-analysis was done in accordance with the PRISMA criteria [19].

### Data extraction and statistical analysis

The primary endpoint was all-site cancer events (incident or, if this was all that was presented, mortal only). The secondary endpoints were all-site cancer incidence (i.e. omitting studies that only reported mortality), all-site cancer mortality and, for those cancers that could present in both sexes, site-specific cancer events, site-specific incidence and site-specific mortality. In sensitivity analysis we also compared all-site cancer incidence and mortality when restricting to the studies that reported both.

The primary metrics were the pooled adjusted RRs and the women-to-men ratios of RRs (RRRs) for individuals with diabetes vs those without diabetes. For each study, we extracted the sex-specific RRs and 95% CIs for individuals with diabetes vs those without diabetes, from which we estimated the RRRs and 95% CIs. To include the largest set of individuals and cancer endpoints, studies that reported either age-adjusted or multiple-adjusted (maximum-available-adjusted, i.e. the maximum set of adjustments available for each study) results were included in our primary analyses. In pooling multiple-adjusted results, the set of adjustments made were allowed to vary by study, but had to include at least one other risk factor for cancer, in addition to age [15, 16]. We obtained pooled estimates of sex-specific RRs across studies using random-effects meta-analyses applied on the log_*e*_ scale. Individual studies were weighted according to the inverse variance of log_*e*_ RRs. The same method was used to pool the RRRs.

The *I*^2^ statistic was used to estimate the percentage of variability across studies due to between-study heterogeneity and the Q test was used to assess whether there was a significant lack of homogeneity. The possibility of publication bias was explored using funnel plots and Egger’s and Begg’s tests. Random-effects meta-regression analyses were used to test for differences between pre-assigned subgroups: study region (Asia or Non-Asia), year of baseline study (pre-1985 or 1986 onwards, and also examined as a continuous variable), ascertainment of diabetes (self-reported only or others), type of diabetes (type 1 or type 2, where studies which did not differentiate type were classified as type 2), level of adjustment (age-adjusted or multiple-adjusted), and study quality (the Newcastle–Ottawa Scale [20] [ESM Table 2], ≥7 or <7 points, and also examined as a continuous variable). Post hoc, we also considered absolute risk difference, examined as a categorical and continuous variable) (ESM Table 3). A *p* value of below 0.05 was considered to be statistically significant in analyses for the primary analyses, i.e. all-site cancer. As many statistical tests were envisaged, a *p* value of below 0.01 was taken to denote significance for site-specific cancers. All analyses were performed using Stata software (release 13; StataCorp, College Station, TX, USA).

## Results

**Fig. 1:**
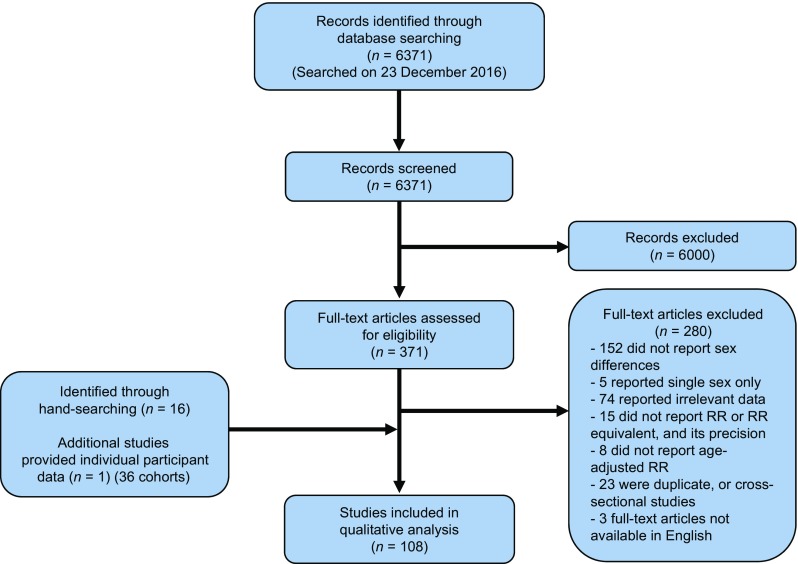
Flow chart of study selection

Of the 6371 articles identified through the systematic search, 371 articles qualified for full-text evaluation, and 107 articles provided summary data on the association between diabetes and the risk of cancer for both sexes [21–127]. In addition, 36 cohorts with individual participant data from the APCSC were included (Fig. 1).

Characteristics of the studies that reported the association between diabetes and all-site cancer incidence or mortality are shown in Table 1 and ESM Table 4. Data on all-site cancer were available from 47 studies, involving 121 cohorts, 19,239,302 individuals (not counting one study [25] that did not state the total number of participants), and 1,082,592 events (not counting one study [65] that did not state the total number of cancer events).

**Table 1:** Characteristics of the studies reporting on the association between diabetes and all-site cancer

Cohort	Country	Baseline study (years)	Follow-up (years)	No. participants (% women)	Mean age (years)	No. with diabetes (% women)	Type of diabetes	Ascertainment of diabetes	No. with outcome (% women)	I or M	Maximum adjustment available
Ragozzino et al [21]	USA	1945–1969	8.6	1135 (NR)	NR	1135 (NR)	Both	Measured	120 (47)	I	Age
Sasazuki et al [22]	Japan (8 cohorts)	1984–1994	9.9	339,459 (54)	35–103	NR	Both	Self-reported	33,022 (40)	I	Age, area, Hx of cerebrovascular disease, CHD, smoking, alcohol consumption, BMI, physical exercise, green leafy vegetable consumption, coffee intake
Gini et al [23]	Italy	2002–2009	3.7	32,247 (45)	65	32,247 (45)	T2	Hospital discharge diagnosis, exemption from medical charges, prescription	2069 (37)	I	Age, year at cancer diagnosis
Berger et al [24]	Denmark	1996–2011	12.6	4,826,142 (50)	41.4	65,690 (47)	Both	Discharge diagnosis, claimed prescription	423,942 (51)	I	Age
Carstensen et al [25]	Australia, Denmark, Finland, Scotland, Sweden	1987–2000	8–38	NR 3,932,900 person-years (50)	NR	NR	T1	Diabetes registry, impatient dataset	9149 (56)	I	Age, date of follow-up, date of birth
Diabetes II-to-Cancer [26]	Germany	2003	3.3	26,742 (53)	64	26,742 (53)	T2	Physician’s diagnosis	1364 (44)	I	Age
VHM&PP Study Cohort [27]	Austria	1988–2001	8.4	140,813 (55)	43	4758 (48)	Both	Measured	5212 (46)	I	Age (stratified), smoking, occupation, BMI
Jee et al [28]	Korea	1992–1995	10	1,298,385 (36)	46.9	62,924 (33)	Both	Self-reported, measured	I: 53,833 (30) M: 26,473 (22)	I, M	Age, smoking, alcohol use
Wang et al [29]	China	2007–2013	6	327,268 (50)	59.8	327,268 (50)	T2	Diabetes registry	7435 (45)	I	Age, urbanisation level
Hsu et al [30]	Taiwan	2000–2007	5.9	14,619 (53)	50.2	14,619 (53)	T1	National health insurance research database	760 (44)	I	Age, calendar year
Adami et al [31]	Sweden	1965–1983	5.2	51,008 (55)	NR	51,008 (55)	Both	Hospital discharge diagnosis	2417 (54)	I	Age
Dankner et al [32]	Israel	2002	11	2,186,196 (53)	21–89	159,104 (53)	Both	Diabetes registry	128,720 (50)	I	Age, ethnic origin, socioeconomic status
NIH-AARP Diet and Health Study [33]	USA	1995–1996	11	494,867 (40)	62.5	44,726 (33)	Both	Self-reported	82,251 (32)	I	Age, BMI, race/ethnicity, education, marital status, family Hx of cancer, self-reported health status, intake of red meat, white meat, fruits, vegetables, alcohol, and coffee, physical activity, smoking, multivitamin use
Xu et al [34]	China	2004	3.7	36,379 (56)	59	36,379 (56)	T2	Diabetes registry	1205 (53)	I	Age
DRT [35]	Austria	2005	8.7	5709 (47)	57.4	5709 (47)	T2	Diabetes registry	525 (45)	I	Age, period in 5 year period groups
NDSS (T2DM) [36]	Australia	1997	5.8	872,706 (47)	60.4	872,706 (47)	T2	Diabetes registry	I: 70,406 (38) M: 26,333 (37)	I, M	Age, calendar year
NDSS (T1DM) [36]	Australia	1997	12	80,676 (48)	27.4	80,676 (48)	T1	Diabetes registry	I: 2079 (50) M: 593 (46)	I, M	Age, calendar year
Walker et al [37]	UK	2001–2007	7	80,838 (45)	55–79	80,838 (45)	T2	Diabetes registry	4285 (43)	I	Age, socioeconomic status
MHS registry [38]	Israel	2000	8	100,595 (53)	61.6	16,721 (47)	Both	Healthcare service database	8977 (43)	I	Age, region, socioeconomic status, use of healthcare services a year prior to index date, BMI, Hx of CVD
CLUE II [39]	USA	1989	17	18,280 (57)	51.8	599 (56)	Both	Self-reported	I: 2481 (52), M: 907 (50)	I, M	Age, education, BMI, smoking, HT treatment, high cholesterol treatment, menopausal status (for women), Hx of use of oral contraceptives (for women), Hx of use of hormone replacement therapy (for women)
Zhang et al [40]	China	2002–2008	6	7950 (52)	61.1	7950 (52)	T2	Diabetes registry	366 (47)	I	Age
Västerbotten Intervention Project [41]	Sweden	2003	8.3^a^	68,301 (51)	46.1^a^	NR^b^	Both	Measured	2669 (53)	I	Age, year of recruitment, smoking
ARIC [42]	USA	1990–1992	15	12,792 (55)	56.9	1125 (56)	Both	Self-reported, prescription	I: 2657 (45) M: 887 (42)	I, M	Age, race/ethnicity, ARIC study site, education, smoking status, cigarette-years smoked, BMI, waist circumference, postmenopausal hormone use (for women)
Wideroff et al [43]	Denmark	1977–1989	5.7	109,581 (50)	Ma: 64 F: 69	109,581 (50)	Both	Hospital discharge diagnosis	8831 (47)	I	Age, calendar year
APCSC (Asia) [18]	Asia (26 cohorts)^c^	1961–1993	7	89,468 (46)	45	4621 (45)	Both	Self-reported, measured	1800 (33)	M	Age, BMI, education, alcohol, smoking
APCSC (Australia and New Zealand) [18]	Australia, New Zealand (9 cohorts)	1989–1996	7	82,913 (52)	51	3365 (44)	Both	Self-reported, measured	2563 (41)	M	Age, BMI, education, alcohol, smoking
Singapore Chinese Health Study [44]	Singapore	1999	10.1	7388 (52)	62	510 (47)	T2	Measured	388 (NR)	M	Age, dialect, interview year, education, smoking, alcohol, BMI
Poole Diabetes Study [45]	UK	1996–1998	5.25	736 (NR)	Ma: 62.9 F: 65.9	368 (NR)	T2	Diabetes registry	45 (58)	M	Age (matched)
DERI Mortality Study [46]	Japan	1965–1979	24.4	1385 (60)	8.8	1385 (60)	T1	Diabetes registry	2 (50)	M	Age
Diabetes UK cohort study [47]	UK	1972–1993	28	T1:23,326 (NR) T2: 5040 (NR)	NR	23,326 (NR)	T1:23,326 (NR) T2: 5040 (NR)	Diabetes registry	T1: 89 (48), T2 185 (32)	M	Age, calendar year, country^d^
JPHC [48]	Japan	1990, 1993	17.8	99,584 (54)	50.2	4286 (36)	Both	Self-reported	5288 (36)	M	Age, BMI, alcohol intake, smoking, Hx of hypertension, physical activity, area (stratified)
Fresco study [49]	Spain (pool of 12 cohorts)	1991	10	55,283 (54)	56	8627 (47)	Both	Self-reported, measured	850 (36)	M	Age, smoking, BMI, SBP, TC, HDLC
NHIS-NSC [50]	Korea	2002–2003	9.7	29,807 (48)	NR	29,807 (48)	T2	National health insurance database	1759 (33)	M	Age
DECODE study [51]	Denmark, Finland, Italy, the Netherlands, Poland, Sweden, UK (17 cohorts)	1966–2004	15.8	44,655 (41)	53.4	3759 (48)	Both	Measured, self-reported	3235 (27)	M	Age, cohort, BMI, total cholesterol, BP, smoking
Tseng [52]	Taiwan	1995–1998	4.4	256,036 (54)	61.2	256,036 (54)	Both	National health insurance database	8098 (41)	M	Age
Piemonte Diabetes Register, Turin Population Register [53]	Italy	1991–1999	7.7	906,065 (NR)	20-	T1: 1608 (NR) T2 29,656 (NR)	Both	Diabetes registry	26,251 (44)	M	Age, area of birth
Hisayama [54]	Japan	1988	16.9	2438 (57)	57.6	298 (45)	Both	Measured, self-reported	229 (37)	M	Age, BMI, total cholesterol, smoking, alcohol, family Hx of cancer, physical activity, dietary factors (daily intakes of total energy, total fat, salt, vitamin A, vitamin B1, vitamin B2, vitamin C, dietary fibre)
Forssas et al [55]	Finland	2003	5	5,147,349 in 1997, 5,300,484 in 2007	1–79	171,596 (54) in 1997 284,832 (49) in 2007	Both	Diabetes registry	54,461 (48)	M	Age^e^
Fedeli et al [56]	Italy	2008	3	167,621 (45)	30–89	167,621 (45)	Both	Archives from subjects exempt from medical charges	5110 (35)	M	Age
HSE, SHeS [57]	UK	1994, 1995	17, 16	204,533 (55)	47	7199 (48)	Both	Self-reported, prescription	5571 (NR)	M	Age, smoking, BMI
Shen et al [58]	China	1998–2001	10.9	66,813 (66)	65-	9225 (66)	Both	Self-reported	6336 (55)	M	Age, alcohol use, smoking, exercise, housing and monthly expenditure, BMI
Weiderpass et al [59]	Sweden	1965–1983	6.7	144,427 (NR)	Ma: 61.3 F: 65.8	144,427 (NR)	Both	Hospital discharge diagnosis	9661 (49)	M	Age, calendar year, comorbidity
CPS II [60]	USA	1982	26	1,053,831 (56)	NR	52,655 (50)	Both	Self-reported	120,221 (46)	M	Age, education, BMI, smoking, alcohol, vegetable intake, red meat intake, physical activity, aspirin use
Verona Diabetes Study [61]	Italy	1987	10	7148 (53)	67	7148 (53)	Both	Medical records, drug prescription database	641 (41)	M	Age
Sievers et al [62]	USA	1975	10	5131 (52)	15-	1266 (58)	T2	Measured	40 (50)	M	Age
2001 ENTRED study [63]	France	2001	5	9101 (NR)	66	9101 (NR)	Both	Self-reported	380 (NR)	M	Age
Allegheny County Type 1 Diabetes Registry [64]	USA	1965–1979	32.9	1075 (47)	10.9	1075 (47)	T1	Medical records	10 (NR)	M	Age, race
BRFSS [65]	USA	1992	5	9074 (NR)	18-	392 (NR)	Both	Self-reported	NR	M	Age
Wong et al [66]	UK	1985	5	4186 (49)	15-	4186 (49)	Both	Diabetes registry	131 (48)	M	Age
Bruno et al [67]	Italy	1988	5.7	1967 (57)	66.5	1967 (57)	T2	Medical record, prescription, sale records of reagent strips and syringes	107 (51)	M	Age, calendar period
Shaw et al [68]	Mauritius, Fiji, Nauru	1980, 1982, 1987	5	9179 (NR)	40.7	595 (53)	Both	Self-reported	97 (57)	M	Age, ethnicity, smoking^f^
Moss et al [69]	USA	1980	8.5	1772 (NR)	66.7	1772 (NR)	Both	Medical records	85 (55)	M	Age
Takayama study [70]	Japan	1992	6.9	29,079 (54)	54.6	1217 (35)	Both	Self-reported	653 (39)	M	Age, smoking, BMI, physical activity, years of education, Hx of HT, intake of total energy, vegetables, fat and alcohol
Chicago Heart Association Detection Project in Industry [71]	USA	1967–1973	12	20,755 (42)	35–64	643 (34)	Both	Self-reported	513 (38)	M	Age, BMI, smoking, SBP, serum cholesterol, education, treatment for HT

If mean values of age or follow-up year were unavailable, median or range was extracted

Wideroff et al was not included in meta-analysis as they did not provide sufficiently accurate CIs for RRs

Studies by Hsu et al, Adami et al, Walker et al, and the Japan Public Health Center-based prospective study, National Diabetes Services Scheme (type 1 diabetes), Takayama study and Västerbotten Intervention Project were excluded from the meta-analysis of primary outcome (all-site cancer) and included in either of the meta-analyses of all-site cancer incidence or mortality only, because of the overlapping of individuals with other studies

^a^Derived from total cohort

^b^Proportion with fasting glucose in the diabetic range (>6.9 mmol/l) was 2% for women and 3% for men

^c^Korean Medical Insurance Corporation cohort was excluded

^d^For type 1 diabetes, RRs for non-South Asians were extracted

^e^RRs for non-insulin-treated diabetes were extracted

^f^RRs for known diabetes were extracted

ARIC, Atherosclerosis Risk in Communities; BRFSS, Behavioral Risk Factor Surveillance System; CLUE II, Give Us a Clue to Cancer and Heart Disease; CPS II, Cancer Prevention Study II; CVD, cardiovascular diseases; DECODE, Diabetes Epidemiology: Collaborative analysis of Diagnostic criteria in Europe; DERI, Diabetes Epidemiology Research International; DRT, Diabetes Registry Tyrol; F, female; HDLC, HDL-cholesterol; HSE, Health Survey for England; HT, hypertension; Hx, history; I, incidence; JPHC, Japan Public Health Center-based prospective study; M, mortality; Ma, male; MHS, Maccabi Healthcare Services; NDSS, National Diabetes Services Scheme; NIH-AARP, National Institutes of Health-American Association of Retired Persons; NHIS-NSC, Korean National Health Insurance Service-National Sample Cohort; NR, not reported; SBP, systolic BP; SHeS, Scottish Health Survey; T1(DM), type 1 diabetes; T2(DM), type 2 diabetes; TC, total cholesterol; VHM&PP, The Vorarlberg Health Monitoring and Promotion Programme; 2001 ENTRED study, 2001–2006 National representative sample of people with diabetes study

**Fig. 2:**
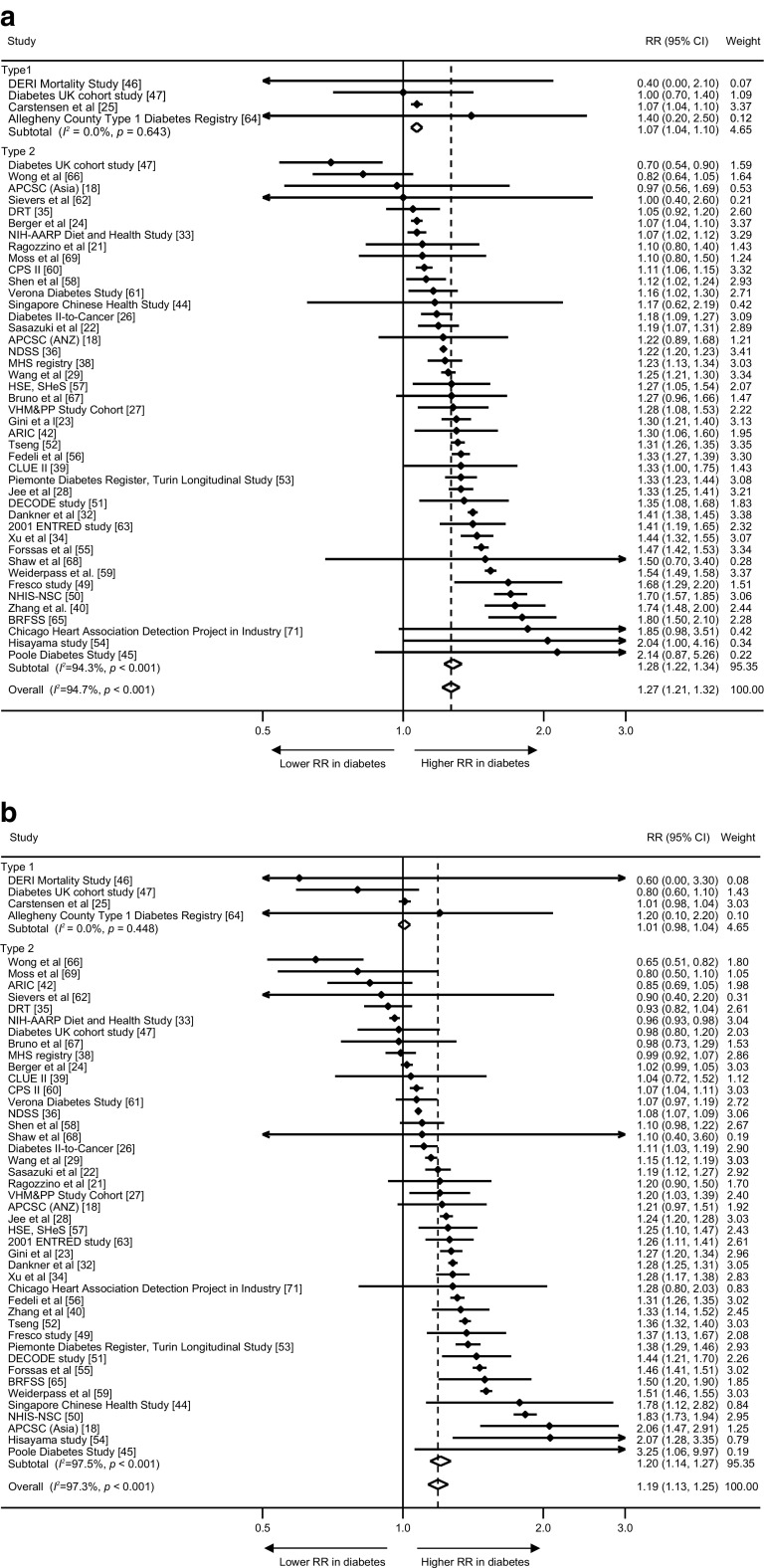
Maximum-available-adjusted RR for all-site cancer, comparing individuals with diabetes with those without diabetes by sex: (**a**) women; and (**b**) men. ANZ, Australia and New Zealand; ARIC, Atherosclerosis Risk in Communities; BRFSS, Behavioral Risk Factor Surveillance System; CLUE II, Give Us a Clue to Cancer and Heart Disease; CPS II, Cancer Prevention Study II; DECODE, Diabetes Epidemiology: Collaborative analysis of Diagnostic criteria in Europe; DERI, Diabetes Epidemiology Research International; DRT, Diabetes Registry Tyrol; 2001 ENTRED study, 2001–2006 National representative sample of people with diabetes study; HSE, Health Survey for England; MHS, Maccabi Healthcare Services; NDSS, National Diabetes Services Scheme; NIH-AARP, National Institutes of Health-American Association of Retired Persons; NHIS-NSC, Korean National Health Insurance Service-National Sample Cohort; SHeS, Scottish Health Survey; VHM&PP, The Vorarlberg Health Monitoring and Promotion Programme

**Fig. 3:**
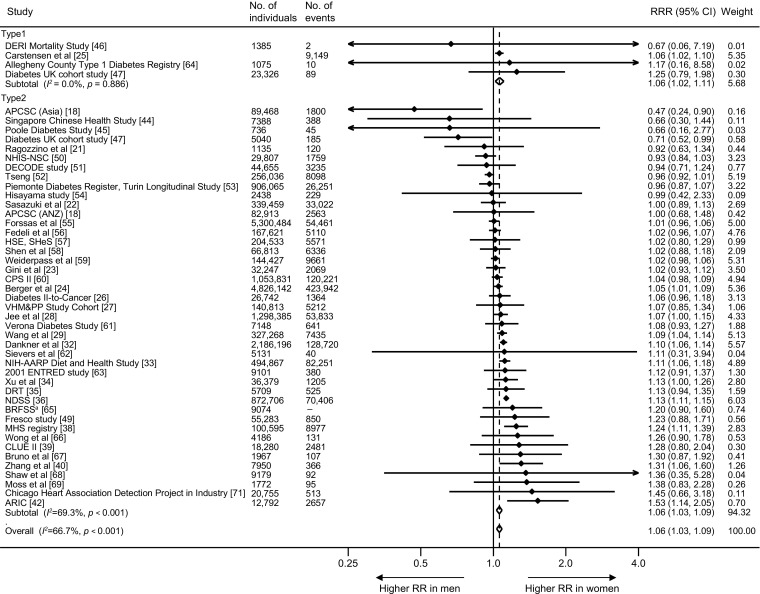
Maximum-available-adjusted women-to-men RRR for all-site cancer, comparing individuals with diabetes with those without diabetes. For definition of study acronyms, please refer to Fig. 2 legend. ^a^The BRFSS did not report the total number of cancer events

**Fig. 4:**
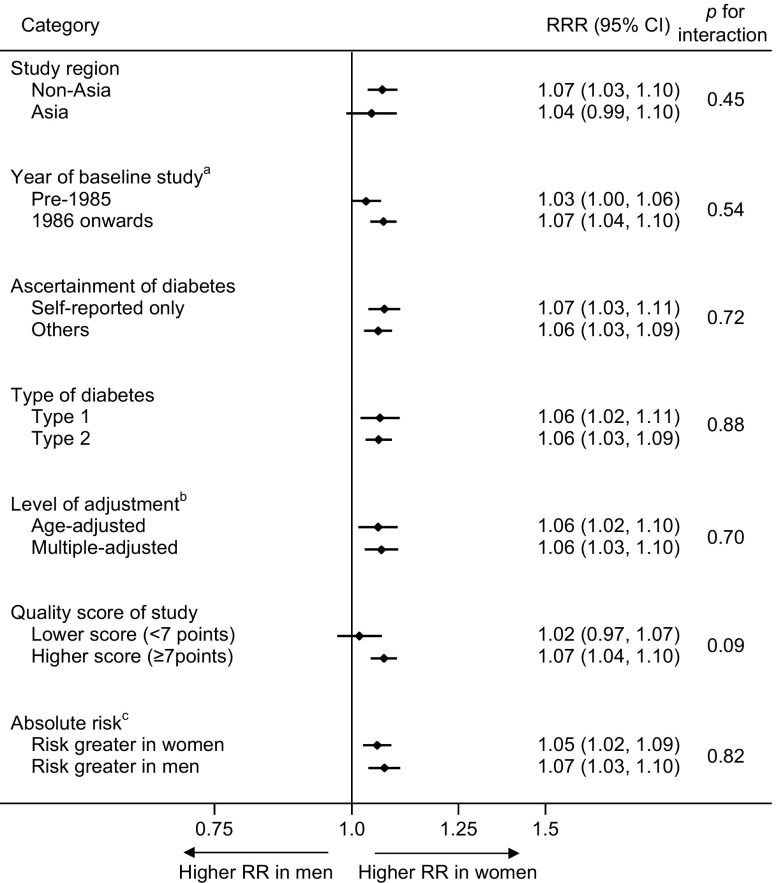
Subgroup analyses of women-to-men RRR for all-site cancer, comparing individuals with diabetes with those without diabetes. ^a^Six studies were excluded because the baseline year bridged over 1985 (i.e. included both pre-1985 and 1986 onwards). ^b^Results using multiple adjustment were used when available and age-adjusted otherwise, as in Fig. 3. ^c^Ten studies were excluded because absolute risks for men and women were unavailable

The maximum-available-adjusted pooled sex-specific RR estimates for combined fatal and non-fatal cancer associated with diabetes were 1.27 (95% CI 1.21, 1.32, *p* < 0.001) for women and 1.19 (1.13, 1.25, *p* < 0.001) for men (Fig. 2). The pooled women-to-men RRR was 1.06 (1.03, 1.09, *p* < 0.001, Fig. 3). The *I*^2^ statistic for heterogeneity between studies was 66.7%, with no evidence of publication bias (Egger’s test *p* = 0.13, Begg’s test *p* = 0.16, ESM Fig. 1). The corresponding RRR was 1.06 (1.02, 1.11, *p* = 0.005) for type 1 diabetes and 1.06 (1.03, 1.09, *p* < 0.001) for type 2 diabetes, without evidence of significant heterogeneity by type of diabetes (*p* for interaction = 0.88, Fig. 4). Exclusion of 22 studies that provided only age-adjusted results had no appreciable effect on the pooled RR estimates (multiple-adjusted pooled RR in women 1.25 [1.17, 1.34], *p* < 0.001, RR in men 1.20 [1.11, 1.29], *p* < 0.001, RRR 1.06 [1.03, 1.10], *p* < 0.001, *I*^2^ = 48.9%) (ESM Figs 2 and 3).

The pooled RRR did not vary substantially by study region (*p* = 0.45), year of baseline study (*p* = 0.54 for categorical analysis, *p* = 0.18 for continuous analysis), ascertainment of diabetes (*p* = 0.72), level of adjustment (*p* = 0.70), quality of study (*p* = 0.09 for categorical analysis) or absolute risk difference between men and women (*p* = 0.82 for categorical analysis, *p* = 0.99 for continuous analysis), with the exception of continuous analysis for quality of study, *p* = 0.01) (Fig. 4 and ESM Fig. 4).

Secondary analyses of incidence (fatal or not) and mortality alone for all-site cancer are described in the ESM. The pooled women-to-men RRR for incidence was 1.10 (1.07, 1.13, *p* < 0.001) (ESM Fig. 5) and for mortality was 1.03 (0.99, 1.06, *p* = 0.16) (ESM Fig. 6).

In sensitivity analysis using only those studies which provided the RRs for both incidence and mortality, the pooled maximum-available-adjusted RRR was 1.12 (1.06, 1.17, *p* < 0.001) for all-site cancer incidence, and 1.10 (1.00, 1.21, *p* = 0.04) for all-site cancer mortality (ESM Fig. 7).

**Fig. 5:**
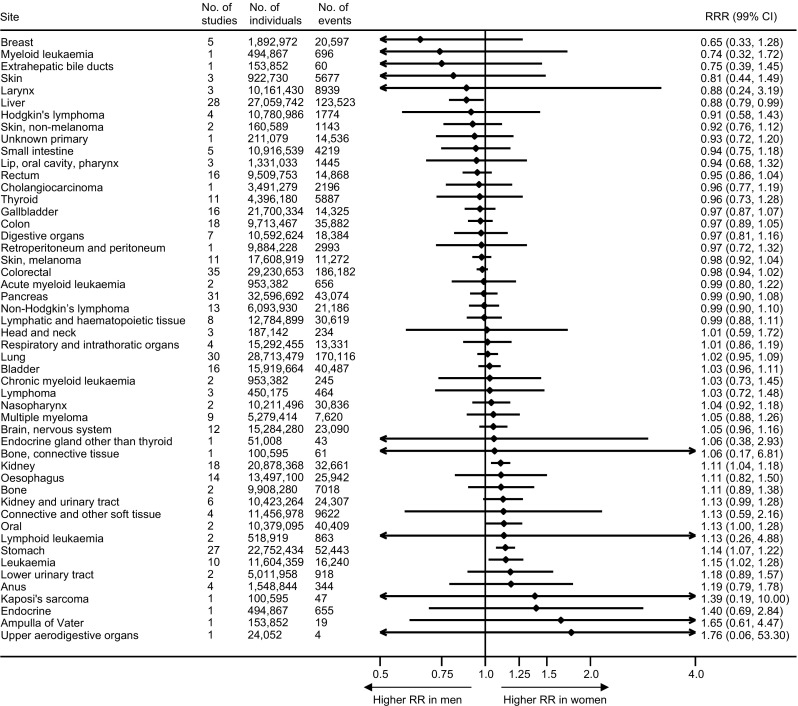
Maximum-available-adjusted pooled women-to-men RRR for cancer at each site, comparing individuals with diabetes with those without diabetes

Data on site-specific cancer were available for 50 sites (50 sites for incidence and 29 sites for mortality) (https://www.georgeinstitute.org/sites/default/files/esm-table.pdf). Diabetes was associated with an increased risk of cancer in 43 sites in women and 42 sites in men, with a statistically significant increase (*p* < 0.01) in risk for those with diabetes in 20 sites in women and 18 sites in men (ESM Fig. 8). The pooled maximum-available-adjusted RRR was statistically significantly higher in women than men for kidney (1.11 [99% CI 1.04, 1.18], *p* < 0.001), oral (1.13 [1.00, 1.28], *p* = 0.009), stomach cancer (1.14 [1.07, 1.22], *p* < 0.001) and leukaemia (1.15 [1.02, 1.28], *p* = 0.002), whereas it was statistically significantly lower for liver cancer (0.88 [0.79, 0.99], *p* = 0.005) (Fig. 5). Separate results for incidence and mortality by site of cancer are described in the ESM (ESM Figs 5, 6, 9–24).

## Discussion

This systematic review, with meta-analysis, of 121 cohorts including more than 19 million individuals and over one million all-site cancer events, demonstrated that diabetes was associated with a 6% higher excess risk of all-site cancer in women than men. Diabetes was associated with several site-specific cancers and conferred a significantly greater excess risk in women than men for oral, stomach and kidney cancer and for leukaemia, but a lower excess risk for liver cancer. The findings were broadly consistent for incident and fatal cancers and across a wide range of prespecified subgroups.

Our findings are in agreement with a previous meta-analysis, which found that the risk of all-site cancer incidence and mortality was significantly increased in both sexes [4]. However, this previous meta-analysis was about a tenth of the size of the current study, and included single-sex studies, and therefore was not able to reliably quantify sex differences as they could have been explained by differences in methods, confounders adjusted for, and the background risks between studies of women and men alone.

As we found some evidence to suggest that the women-to-men RRRs tended to be smaller in studies of lower quality (Fig. 4 and ESM Fig. 4), our results may underestimate any true sex difference. A significant degree of heterogeneity was also observed between studies conducted in and outside Asia with regards to all-site cancer mortality (ESM Fig. 19). However, we did not find heterogeneity between regions for our primary outcome, nor for the other secondary outcomes (all-site cancer incidence), and thus we speculate that this may be a chance finding consequent to the high number of statistical tests conducted.

Although we found a slightly higher women-to-men RRR for cancer incidence than cancer mortality, the finding may be explained by chance differences between the included studies, as almost identical pooled RRR estimates were obtained in the sensitivity analysis restricted to five studies which provided the sex-specific RRs for both incidence and mortality from the same study.

With regard to cancer at specific sites, previous meta-analyses have yielded inconsistent results of increased (stomach [5], lung [6], kidney [7]), similar (oesophagus [8], colorectum [9], pancreas [10], bladder [11], thyroid [12]) or decreased (liver [13]) excess risk of cancer associated with diabetes in women compared with men. However, unlike our methods, these analyses included single-sex studies as well as studies among both women and men.

There are several possible explanations for the excess risk of cancer conferred by diabetes in women than men. One possible mechanism is poor glycaemic control in women with diabetes compared with men with diabetes [128, 129]. Hyperglycaemia may have carcinogenic effects by causing DNA damage [130], which could result from increased oxidative stress due to hyperglycaemia [130] or from hyperglycaemia itself [131]. Historically, women were likely to be undertreated or receive less intensive care compared with men [128, 132]. Further, a recent study showed that adherence to glucose-lowering medication was lower in women than men [133]. As such, the carcinogenic effects of hyperglycaemia may be enhanced in women and subsequently lead to an increased cancer risk compared with men. Alternatively, cumulative exposure to insulin resistance and subsequent hyperinsulinaemia may be longer in women compared with men. The average duration of impaired glucose tolerance or impaired fasting glucose has been found to be more than 2 years longer in women than men [134], suggesting that women may have more exposure to, often untreated, hyperinsulinaemia in the prediabetic state. Hyperinsulinaemia promotes cancer cell proliferation by stimulating the insulin receptor directly and insulin-like growth factor-1 indirectly [135]. Another factor that may, to some extent, explain the smaller RR for incidence of all-site cancer in men compared with women is the apparent protective effect of diabetes on prostate cancer in men with diabetes [136]. Sex-specific cancers or site-specific cancers in which diabetes conferred greater or lower excess risk in women than men may also account for the association, although the degree of contribution cannot be determined from our analyses. In addition to sex difference for all-site cancer, we found also that diabetes conferred a significantly greater RR in women than men for oral, stomach and kidney cancer and for leukaemia, but a lower RR for liver cancer. The underlying mechanisms for sex differences in each specific association are not clear. However, unmeasured confounding factors specific to each site, such as *Helicobacter pylori* infection for stomach cancer [137] and hepatitis virus infection for liver cancer [138], might be involved. However, the literature around mechanisms underpinning the sex differences in site-specific cancers is scant and further studies are required to confirm and clarify these sex differences in site-specific associations. Finally, the studies in our analyses were not adjusted for female-specific factors including pregnancy, menopausal status and use of hormone replacement therapy that have also been associated with diabetes [139] and cancer [140].

We quantified sex differences based on RRs rather than risk differences. This might introduce a statistical artefact, in which the generally higher absolute risk for cancer in men, and the same risk difference subsequent to diabetes in each sex, would translate to a greater relative risk in women than men. However, this would require that risks of cancers associated with diabetes are additive rather than multiplicative, which is not generally considered to be the case in epidemiology. Indeed, RRs are much more commonly reported than risk differences in both epidemiological studies and clinical trials. Also, unlike risk differences, RRs are typically fairly stable across populations with different background risks, which make them suitable for summarisation of effects in meta-analyses. Furthermore, our previous meta-analyses on risk factors for cardiovascular diseases demonstrated that detection of a female disadvantage in RRs is not inevitable when men have higher absolute risk [141, 142]. We thus believe that the use of RRs in the present analyses is both practical and justifiable.

The strengths of this meta-analysis are its size and the inclusion of studies on the sex-specific effects of diabetes on all-site cancer and 50 site-specific cancers, which enabled us to conduct the most comprehensive analyses to date on the sex-specific effects of diabetes on cancer risk. To limit the risk of bias, we only included cohort studies that were conducted in men and women and had adjusted for at least age. Limitations of this study are inherent to the use of published data and the heterogeneity between studies in ascertainment of diabetes, study design and duration, endpoint definition and degree of adjustment for confounders. Nevertheless, a range of subgroup analysis provided broadly consistent results. However, as we compared women and men from within the same study, any effect of differences in methods between studies is likely to have affected women and men similarly. We therefore assume that the sex comparisons reported in this analysis are still valid. Second, the lack of data on duration of diabetes and the degree of glycaemic control precluded more detailed analyses on the effect of diabetes on the risk of cancer. Third, as this meta-analysis largely used published data, endpoint definition varied across the studies. Fourth, in analysis of all-site cancer, the women-to-men RRRs depend not only on the strengths of the RRRs of site-specific cancers (as illustrated by Fig. 5), but also on the relative incidence of site-specific cancers, which varies considerably between populations. This is likely to be a key factor in the high between-study heterogeneity we show in Fig. 3. Finally, studies generally did not adjust for obstetric and gynaecological history and unmeasured confounding is likely in the current estimates. However, confounding is likely to have been non-differentially distributed between women and men from the same study and we therefore assume that it had only a negligible effect on the reported associations.

In conclusion, diabetes is a risk factor for all-site cancer in both sexes, with a stronger effect in women than men. Sex differences varied across the location of the cancer, heightening the importance of a sex-specific approach to quantification of the role of diabetes in cancer research, prevention and treatment. Further studies are needed to clarify the mechanisms underlying the sex differences in the diabetes–cancer association.

## Electronic supplementary material

ESM (PDF 1.26 MB)

## Data Availability

The datasets generated during and/or analysed in the current study are available from the corresponding author on reasonable request.

## Abbreviations

APCSC: Asia Pacific Cohort Studies Collaboration
RRR: Ratio of RR
