# Supplementary material for: Sex differences in the association between diabetes and cancer: a systematic review and meta-analysis of 121 cohorts including 20 million individuals and one million events
Source: Diabetologia. 2018 Jul 20;61(10):2140–54. doi: 10.1007/s00125-018-4664-5 (PMC6133170; doi:10.1007/s00125-018-4664-5)
Supplement: Supplementary file 1 — (PDF 1.26 MB) [file 125_2018_4664_MOESM1_ESM.pdf]

ESM Results

Sex-specific association between diabetes and all-site cancer incidence

Data on all-site cancer incidence were available from 22 studies, 33 cohorts, 10,942,431 individuals (not counting one study [1] which did not state the total number of participants), and 843,865 events. Compared with subjects without diabetes, those with diabetes had a 24% (95% CI 18%, 30%, p <0.001) increased risk of incident cancer in women and 11% (6%, 16%, p <0.001) in men (ESM Fig. 9). The pooled women-to-men RRR for incidence of all-site cancer was 1.10 (1.07, 1.13 p <0.001) (ESM Fig. 5). Across subgroups, the equivalent pooled RRR was 1.06 (1.02, 1.10, p = 0.003) for type 1 diabetes and 1.11 (1.08, 1.13, p <0.001) for type 2 diabetes. There was significant heterogeneity in the diabetes-cancer association between studies (I^2 = 51.3%, p for heterogeneity = 0.003). Exclusion of the 11 studies with only age-adjusted RRs did not change the RR and RRR estimates appreciably (RR in women 1.27 [1.19, 1.36], p <0.001, RR in men 1.11 [1.02, 1.20], p = 0.02, RRR 1.12 [1.08, 1.16], p <0.001, I^2 = 46.2%) (ESM Fig. 10 and 11). There was no evidence of publication bias (Egger's test p = 0.82, Begg's test p = 0.15; ESM Fig. 12).

There was no evidence of between-subgroup heterogeneity in the RRR by study region (p = 0.64), year of baseline study (p = 0.73 for categorical analysis, p = 0.25 for continuous analysis), ascertainment of diabetes (p = 0.72), type of diabetes (p = 0.33), level of adjustment (p = 0.27), or quality of study (p = 0.75 for categorical analysis, p = 0.14 for continuous analysis) (ESM Fig. 13 and 14).

Sex-specific association between diabetes and all-site cancer mortality

Data on all-site cancer mortality were available from 36 studies, 101 cohorts, 10,923,139 individuals, and 309,992 deaths (not counting one study [2] which did not state the total number of cancer). The maximum available-adjusted RR for cancer mortality associated with diabetes was 1.29 (1.21, 1.38, p <0.001) in women and 1.24 (1.15, 1.34, p <0.001) in men (ESM Fig. 15). The pooled maximum available-adjusted women-to-men RRR for all-site cancer mortality was 1.03 (0.99, 1.06, p = 0.16) (ESM Fig. 6). The pooled RRR was 1.12 (0.96, 1.31, p = 0.14) for type 1 diabetes and 1.02 (0.99, 1.06, p = 0.25) for type 2 diabetes, with no evidence of heterogeneity between type of diabetes (p for heterogeneity = 0.32). The pooled RRs and RRR did not change significantly (RR in women 1.28 [1.15, 1.42], p <0.001, RR in men 1.27 [1.16, 1.39], p <0.001, RRR 1.01 [0.96, 1.06] , p = 0.65) after exclusion of the studies which reported only age-adjusted RR (ESM Fig. 16 and 17). There was no evidence of publication bias (Egger's test p = 0.54, Begg's test p = 0.11, ESM Fig. 18).

The pooled RRR did not differ significantly by year of baseline study (p = 0.67 for categorical analysis, p = 0.89 for continuous analysis), ascertainment of diabetes (p = 0.62), type of diabetes (p = 0.32), level of adjustment (p = 0.55), or quality of study (p = 0.18 for categorical analysis, p = 0.19 for continuous analysis). A significant heterogeneity was observed by study region (RRR 1.05 [95%CI 1.01-1.08], p = 0.01 for non-Asia, 0.97 [0.91-1.02], p = 0.23 for Asia, p for interaction = 0.02) (ESM Fig. 19 and 20).

Sex-specific association between diabetes and incidence of cancer at specific sites

The pooled maximum available-adjusted RR in people with diabetes versus those without diabetes was increased in 41 sites in women and in 42 sites in men; amongst these, 21 sites in women and 15 sites in men showed a statistically significant (p <0.01) inceased risk (ESM Fig. 21). The pooled maximum available-adjusted women-to-men RRR for incidence of cancer was significantly greater than one (i.e. affecting women more than men) for kidney (1.11 [99% CI 1.04, 1.19], p <0.001), oral (1.13 [1.01, 1.27], p = 0.009), and stomach (1.16 [1.06, 1.27] , p <0.001) cancer (ESM Fig. 22).

Sex-specific association between diabetes and mortality from cancer at specific sites

Diabetes was associated with an increased risk of cancer at 26 sites in women and 20 sites in men (ESM Fig. 23). Among these, a statistically significant increased risk was observed at 11 sites in women and 12 sites in men. On the other hand, only cancer of the brain and the nervous system in men was associated with a decreased risk in people with diabetes. The pooled maximum available-adjusted women-to-men RRR for mortality from cancer was significantly less than unity for cancer of the breast (RRR 0.28 [99% CI 0.12, 0.66], p <0.001) and liver (0.84 [0.71, 0.99] , p = 0.008) (ESM Fig. 24).

ESM References

[1] Carstensen B, Read SH, Friis S, et al. (2016) Cancer incidence in persons with type 1 diabetes: a five-country study of 9,000 cancers in type 1 diabetic individuals. Diabetologia 59: 980-988

[2] Tierney EF, Geiss LS, Engelgau MM, et al. (2001) Population-based estimates of mortality associated with diabetes: use of a death certificate check box in North Dakota. Am J Public Health 91: 84-92

ESM Table 1. Search strategies

PubMed (Searched on Dec 23, 2016)

No	Search item
1	“Neoplasms” [MeSH Terms]
2	“Neoplasm” [All Fields]
3	“Cancer” [All Fields]
4	“Carcinoma” [All Fields]
5	“Tumor” [All Fields]
6	1 or 2 or 3 or 4 or 5
7	“Diabetes Mellitus” [MeSH Terms]
8	“Diabetes” [All Fields]
9	7 or 8
10	“Men” [MeSH Terms]
11	“Male” [MeSH Terms]
12	“Men” [All Fields]
13	“Male” [All Fields]
14	10 or 11 or 12 or 13
15	“Women” [MeSH Terms]
16	“Female” [MeSH Terms]
17	“Women” [All Fields]
18	“Female” [All Fields]
19	15 or 16 or 17 or 18
20	“Cohort Studies” [MeSH Terms]
21	“Follow Up Studies” [MeSH Terms]
22	“Prospective Studies” [MeSH Terms]
23	“Longitudinal Studies” [MeSH Terms]
24	“Cohort” [All Fields]
25	“Follow-up” [All Fields]
26	“Prospective” [All Fields]
27	“Longitudinal” [All Fields]
28	20 or 21 or 22 or 23 or 24 or 25 or 26 or 27
29	6 and 9 and 14 and 19 and 28

ESM Table 2. Newcastle-Ottawa Quality assessment scale modified from reference 19

A study can be awarded a maximum of one point for each * within the Selection and Outcome categories. A maximum of two point can be given for Comparability.

Selection

S1) Representativeness of the exposed cohort

a) truly representative of the general population*

b) somewhat representative of the general population

c) selected group of users e.g. nurses, volunteers

d) no description of the derivation of the cohort

S2) Selection of the non exposed cohort

a) drawn from the same community as the exposed cohort*

b) drawn from a different source

c) no description of the derivation of the non exposed cohort

S3) Ascertainment of exposure

a) secure record*

b) secure record or written self report

c) written self report

d) no description

S4) Demonstration that outcome of interest was not present at start of study

a) yes*

b) no

Comparability

C1) Comparability of cohorts on the basis of the design or analysis

a) study controls for age*

b) study also controls for additional factors*

Outcome

O1) Assessment of outcome

a) reference to medical records*

b) record linkage*

c) self report

d) no description

O2) Was follow-up long enough for outcomes to occur

a) yes (at least 5 years)*

b) no

O3) Adequacy of follow up of cohorts

a) complete follow up - all subjects accounted for*

b) subjects lost to follow up unlikely to introduce bias - > 90% follow up, or description provided of those lost*

c) follow up rate < 90% and no description of those lost

d) no statement

ESM Table 3. Absolute risks of all-site cancer for the data in Figure 2 and 3

Cohort	Men	Women	Difference (men - women)
Ragozzino et al.	na	na	na
Sasazuki et al.	1.06	0.57	0.49
Gini et al.	1.98	1.43	0.55
Berger et al.	0.68	0.71	-0.02
Carstensen et al.	0.20	0.26	-0.06
Diabetes II-to-Cancer	1.84	1.27	0.57
VHM&PP Study Cohort	0.53	0.36	0.17
Jee et al.	0.46	0.34	0.11
Wang et al.	0.42	0.34	0.08
Dankner et al.	0.56	0.51	0.05
NIH-AARP Diet and Health Study	1.72	1.20	0.52
Xu et al.	0.96	0.85	0.11
DRT	1.13	0.97	0.16
NDSS (T2DM)	1.63	1.12	0.51
MHS registry	1.21	1.01	0.20
CLUE II	0.90	0.72	0.18
Zhang et al.	0.85	0.69	0.16
ARIC	1.97	1.27	0.70
APCSC (Asia)	0.35	0.19	0.16
APCSC (Australia and New Zealand)	0.40	0.25	0.15
Singapore Chinese Health Study	na	na	na
Poole Diabetes Study	0.90	1.49	-0.59
DERI Mortality Study	0.0074	0.0049	0.0025
Diabetes UK cohort study (T1DM, T2DM)	na	na	na
Fresco study	0.21	0.10	0.11
NHIS-NSC	0.79	0.41	0.38
DECODE study	0.57	0.30	0.26
Tseng, et al.	0.91	0.56	0.35
Piemonte Diabetes Register, Turin Population Register	na	na	na
Hisayama	0.86	0.35	0.51
Forssas et al.	0.32	0.27	0.04
Fedeli et al.	1.20	0.79	0.41
HSE, SHeS	na	na	na
Shen et al.	1.16	0.72	0.45
Weiderpass et al.	1.07	0.93	0.14
CPS II	0.54	0.36	0.17
Verona Diabetes Study	1.12	0.70	0.43
Sievers et al.	0.09	0.07	0.01
2001 ENTRED study	na	na	na
Allegheny County Type 1 Diabetes Registry	na	na	na
BRFSS	na	na	na
Wong et al.	0.64	0.61	0.02
Bruno et al.	1.09	0.85	0.23
Shaw et al.	0.20	0.22	-0.02
Moss et al.	na	na	na
Chicago Heart Association Detection Project in Industry	0.22	0.19	0.04

Incidence (or mortality) rates were described as per 100 person-years or (%) per year.

na: not available.

ESM Table 4. Quality assessment of the included studies

Cohort	Scores								
	S1	S2	S3	S4	C1	O1	O2	O3	Sum
Ragozzino et al.	1	0	1	0	1	1	1	0	**5**
Sasazuki et al.	1	1	0	1	2	1	1	0	**7**
Gini et al.	1	1	1	1	1	1	0	1	**7**
Berger et al.	1	1	1	1	1	1	1	1	**8**
Carstensen et al.	1	1	1	1	1	1	1	1	**8**
Diabetes II-to-Cancer	1	0	1	1	1	1	0	1	**6**
VHM&PP Study Cohort	1	1	1	1	2	1	1	1	**9**
Jee et al.	0	1	0	1	2	1	1	1	**7**
Wang et al.	1	0	1	1	2	1	1	0	**7**
Hsu et al.	1	1	1	1	1	1	1	1	**8**
Adami et al.	0	1	1	0	1	1	1	1	**6**
Dankner et al.	1	1	1	1	2	1	1	1	**9**
NIH-AARP Diet and Health Study	1	1	0	1	2	1	1	1	**8**
Xu et al.	1	1	1	0	1	1	0	1	**6**
DRT	1	1	1	0	1	1	1	1	**7**
NDSS (T2DM)	1	1	1	1	1	1	1	1	**8**
NDSS (T1DM)	1	1	1	1	1	1	1	1	**8**
Walker et al.	1	1	1	1	2	1	1	1	**9**
MHS registry	1	1	1	1	2	1	1	1	**9**
CLUE II	1	1	0	1	2	1	1	1	**8**
Zhang et al.	1	1	1	0	1	1	1	1	**7**
Västerbotten Intervention Project	1	1	1	1	2	1	1	1	**9**
ARIC	1	1	0	1	2	1	1	0	**7**
APCSC (Asia)	1	1	0	1	2	1	1	1	**8**
APCSC (Australia and New Zealand)	1	1	0	1	2	1	1	1	**8**
Singapore Chinese Health Study	1	1	1	1	2	1	1	1	**9**
Poole Diabetes Study	1	1	1	0	1	1	1	1	**7**
DERI Mortality Study	1	0	1	0	1	1	1	1	**6**
Diabetes UK cohort study	1	0	1	0	2	1	1	1	**7**
JPHC	1	1	0	1	2	1	1	1	**8**
Fresco study	1	1	0	0	2	1	1	1	**7**
NHIS-NSC	1	0	1	0	1	1	1	1	**6**
DECODE study	0	1	0	0	2	1	1	0	**5**
Tseng, et al.	1	0	1	0	1	1	0	1	**5**
Piemonte Diabetes Register, Turin Population Register	1	1	1	0	2	1	1	1	**8**
Hisayama	1	1	0	1	2	1	1	1	**8**
Forssas et al.	1	1	1	0	1	1	1	1	**7**

Fedeli et al.	1	1	1	0	1	1	0	0	**5**
HSE, SHeS	1	1	0	1	2	1	1	1	**8**
Shen et al.	1	1	0	0	2	1	1	1	**7**
Weiderpass et al.	0	1	1	0	2	1	1	1	**7**
CPS II	1	1	0	1	2	1	1	1	**8**
Verona Diabetes Study	1	1	1	0	1	1	1	1	**7**
Sievers et al.	1	1	1	0	1	1	1	1	**7**
2001 ENTRED study	1	0	0	0	1	1	1	1	**5**
Allegheny County Type 1 Diabetes Registry	1	1	1	0	2	1	1	1	**8**
BRFSS	1	1	0	0	1	1	1	0	**5**
Wong et al.	1	1	1	0	1	1	1	1	**7**
Bruno et al.	1	0	1	0	1	1	1	1	**6**
Shaw et al.	1	1	0	0	2	1	1	1	**7**
Moss et al.	1	1	1	0	1	1	1	1	**7**
Takayama study	1	1	0	1	2	1	1	1	**8**
Chicago Heart Association Detection Project in Industry	0	1	0	0	2	1	1	1	**6**

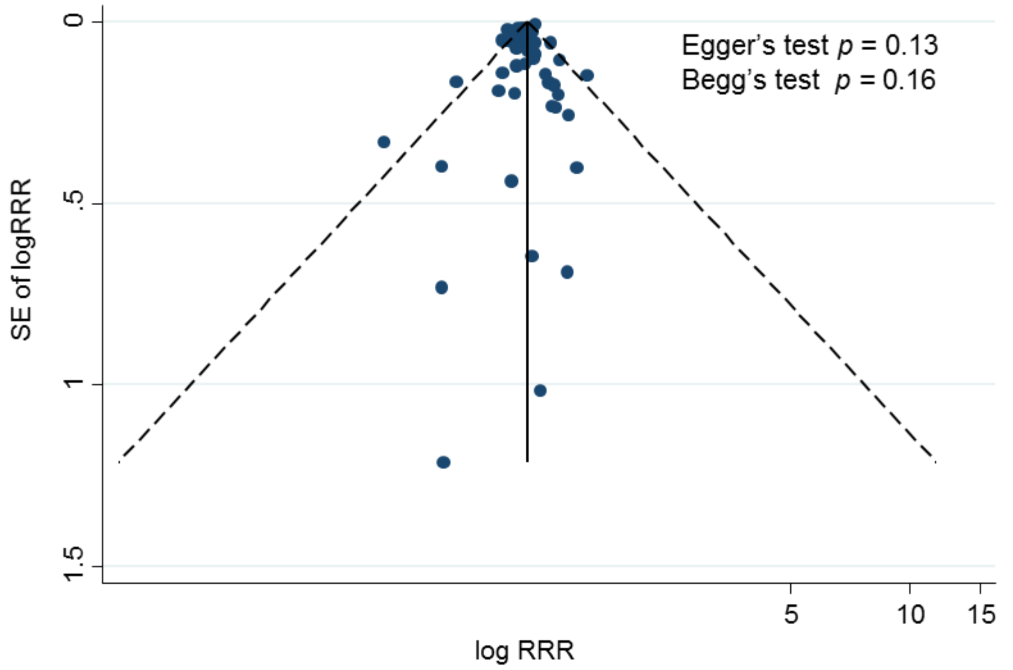

ESM Figure 1. Funnel plot with pseudo 95% confidence limits for the data in Figure 3.

Abbreviations; RRR, ratio of relative risk; SE, standard error.

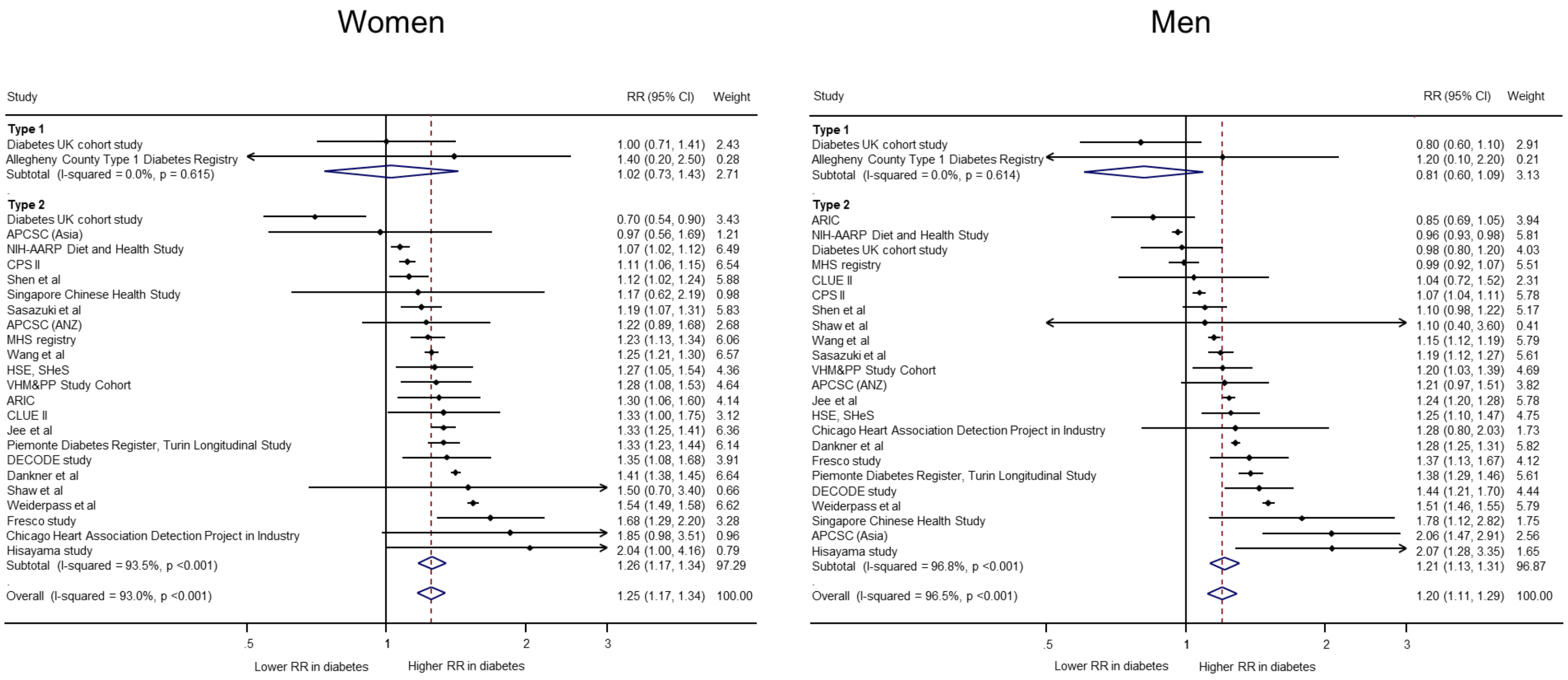

ESM Figure 2. Multiple-adjusted relative risk for all-site cancer, comparing individuals with diabetes to those without diabetes by sex.

Studies that provided only age-adjusted results were excluded from the analysis.

Abbreviations; CI, confidence interval; RR, relative risk.

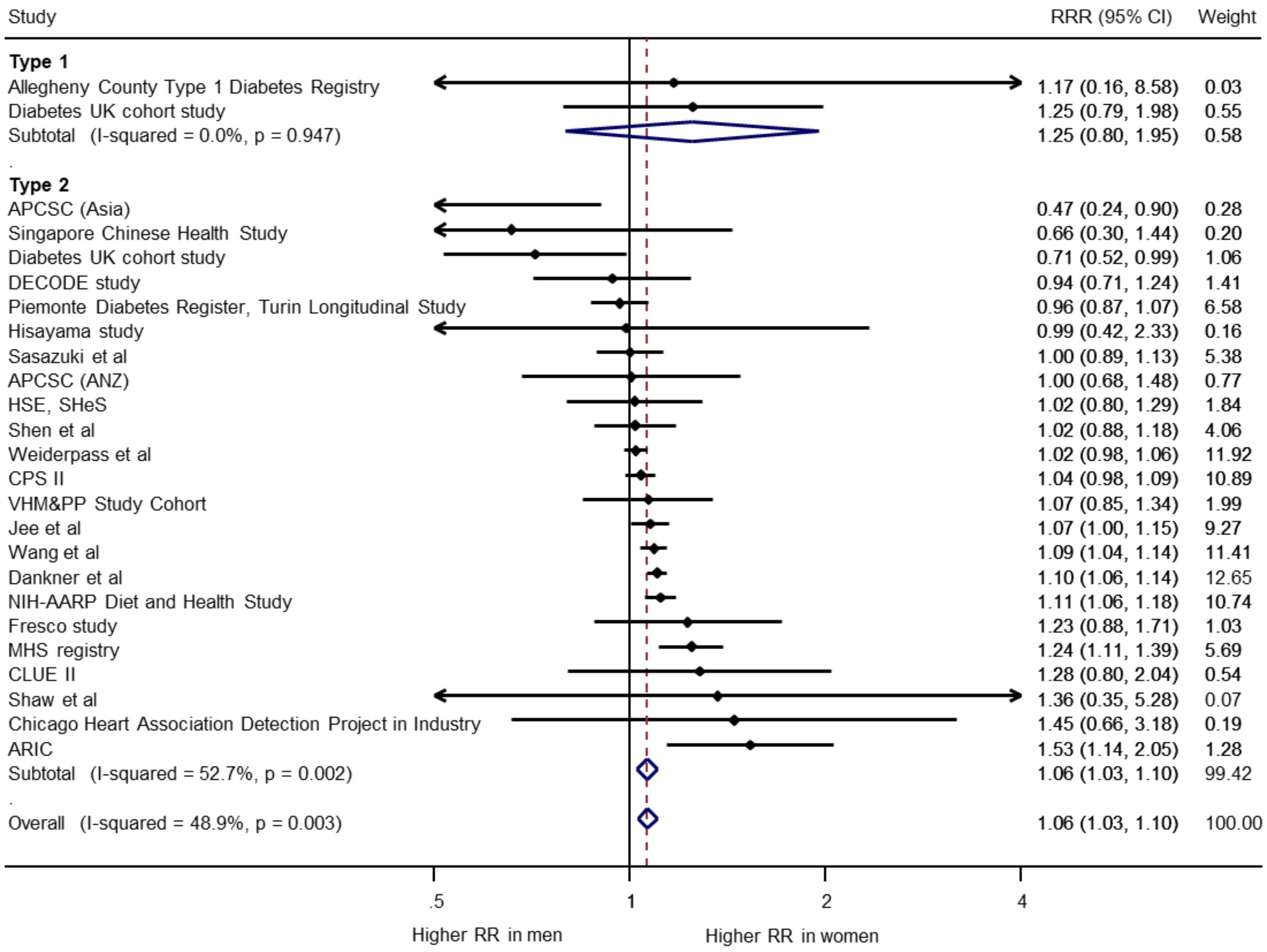

ESM Figure 3. Multiple-adjusted women-to-men ratio of relative risk for all-site cancer, comparing individuals with diabetes to those without diabetes.

Studies that provided only age-adjusted results were excluded from the analysis.

Abbreviations; RR; relative risk; RRR, ratio of relative risk; CI, confidence interval.

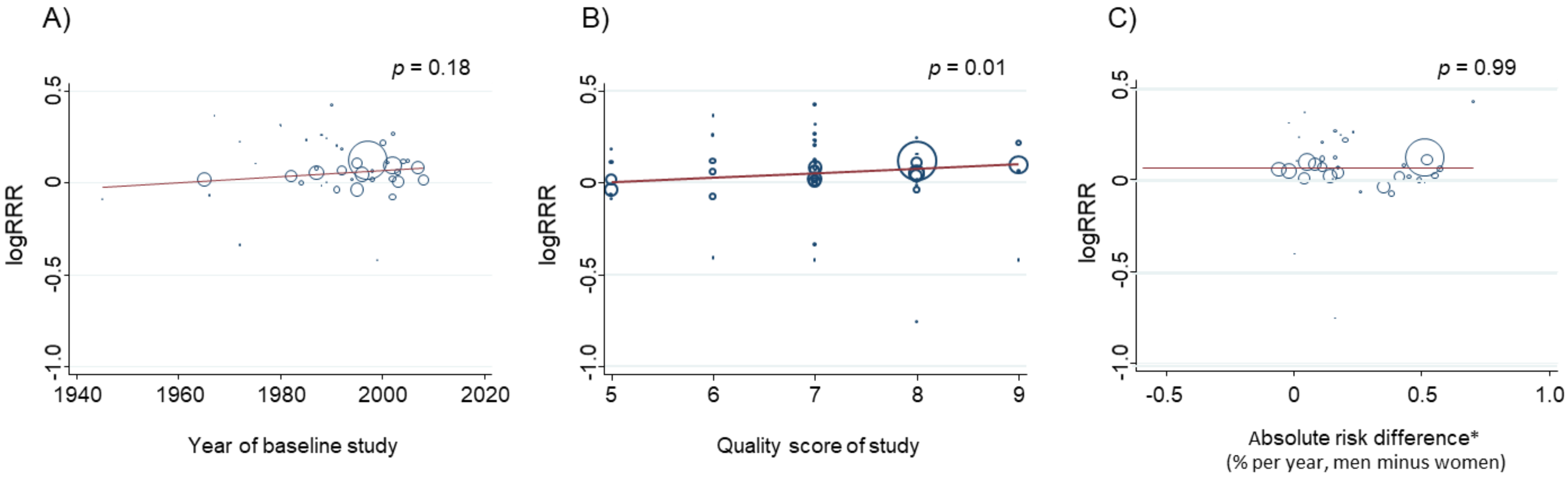

ESM Figure 4. Meta-regression of log (women-to-men ratio of relative risk for all-site cancer) against A) year of baseline study and B) quality score of study (the Newcastle-Ottawa Scale) C) absolute risk difference between men and women for the data in Figure 3.

The circles for each study are drawn in proportion to the inverse variance. Abbreviations; RRR, ratio of relative risk.

* Ten study were excluded because absolute risks of men and women were unavailable.

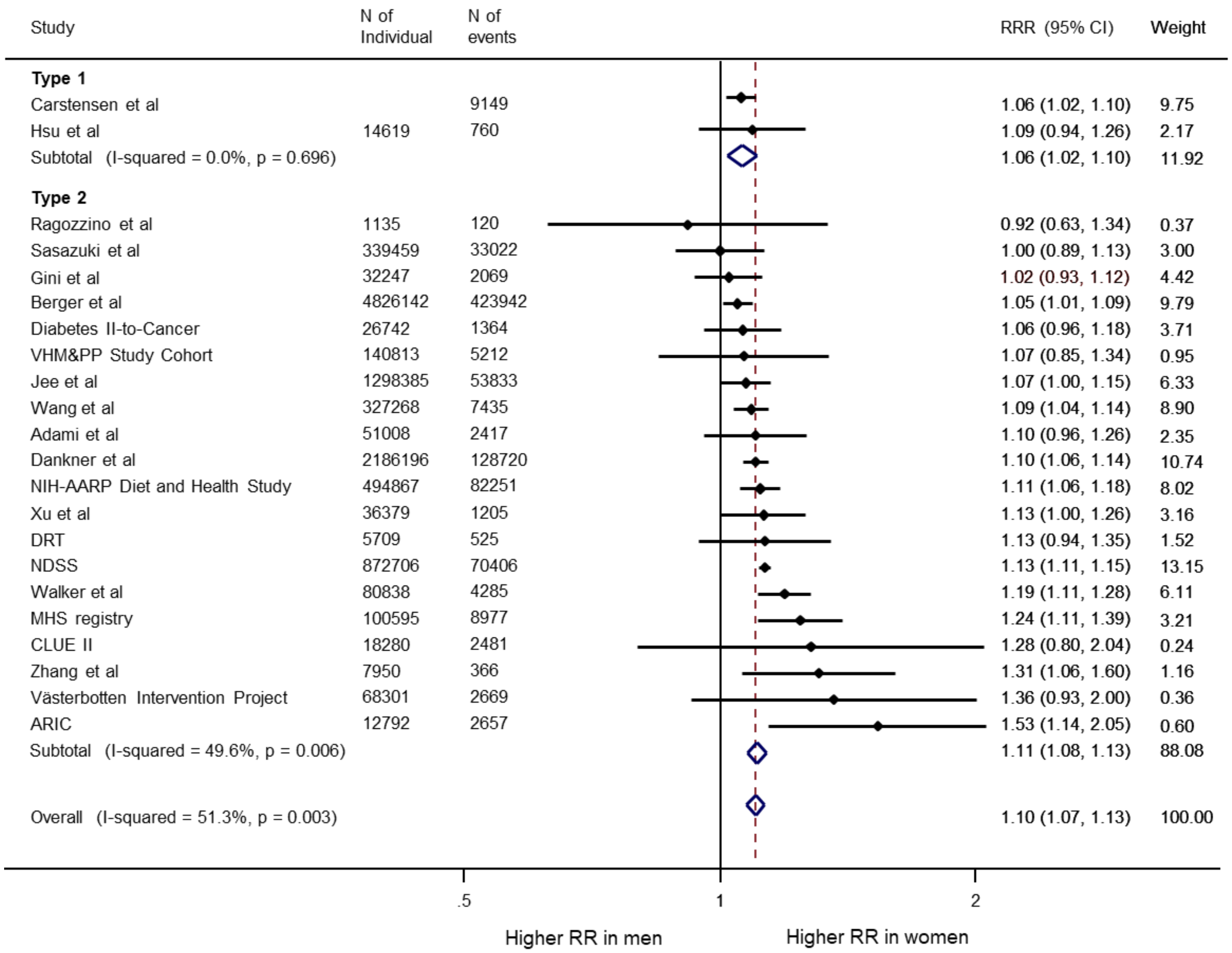

ESM Figure 5. Maximum available-adjusted women-to-men ratio of relative risk for all-site cancer incidence, comparing individuals with diabetes to those without diabetes.

Abbreviations; RR; relative risk; RRR, ratio of relative risk; CI, confidence interval.

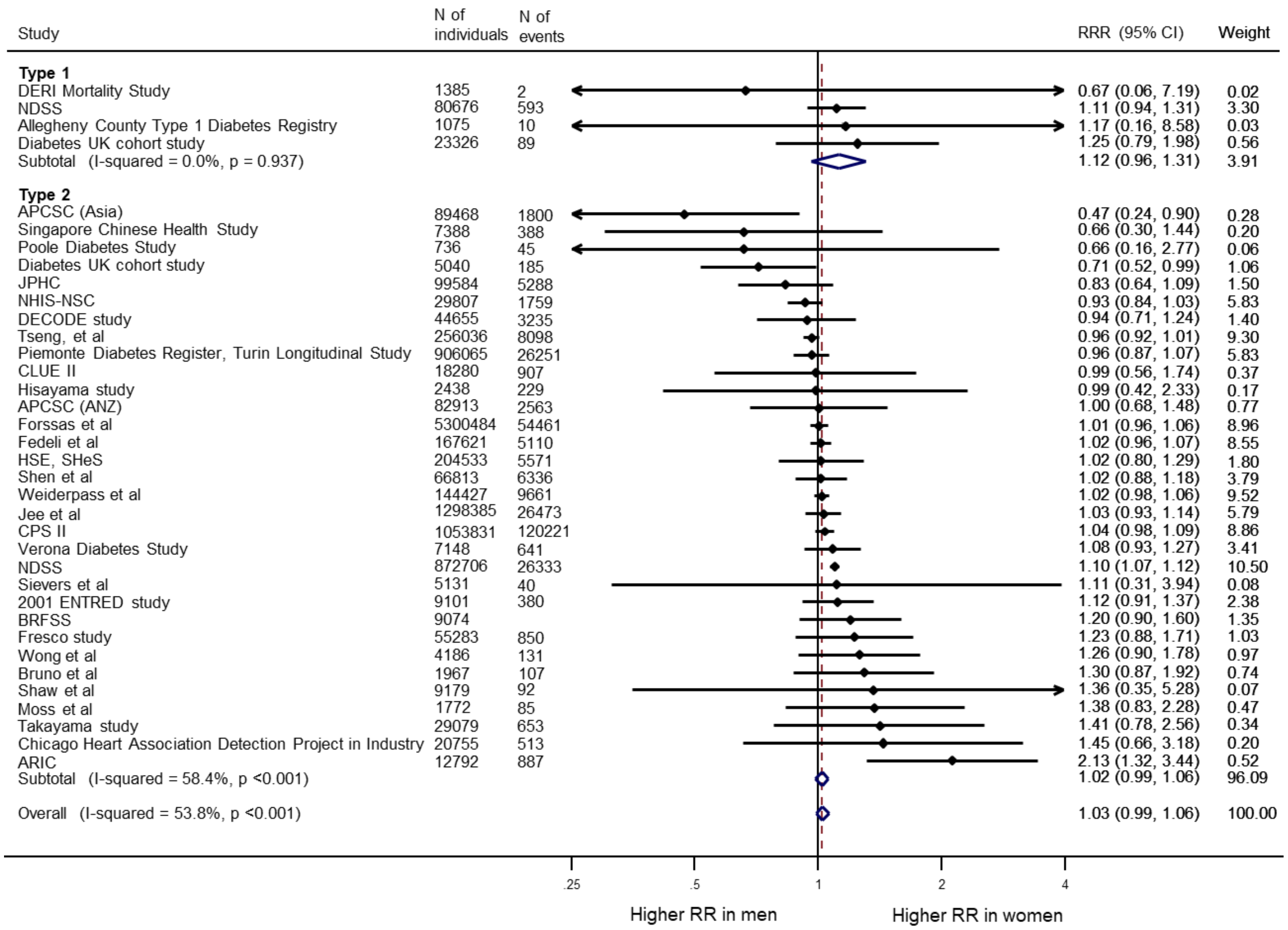

ESM Figure 6. Maximum available-adjusted women-to-men ratio of relative risk for all-site cancer mortality, comparing individuals with diabetes to those without diabetes.

Abbreviations; RR; relative risk; RRR, ratio of relative risk; CI, confidence interval.

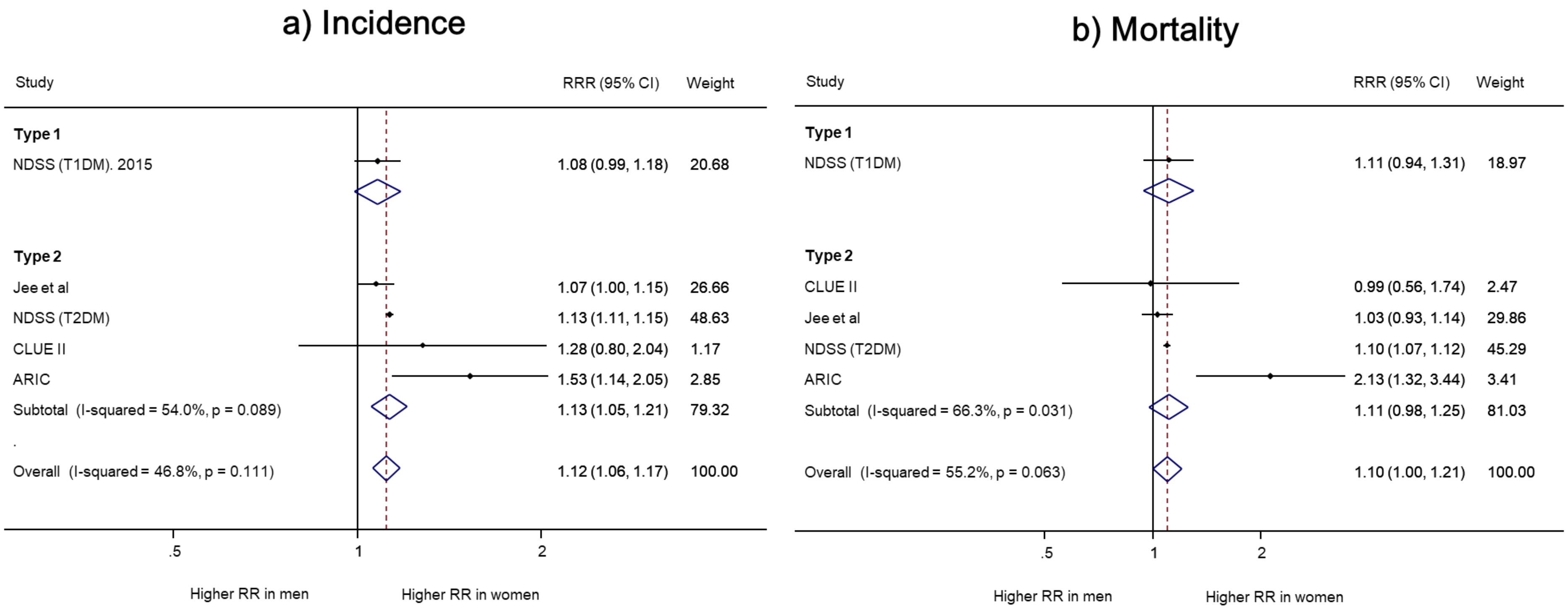

ESM Figure 7. Maximum available-adjusted women-to-men ratio of relative risk for all-site cancer A) incidence and B) mortality for individuals with and without diabetes in studies which provided the results on both incidence and mortality from the same study.

Abbreviations; RR; relative risk; RRR, ratio of relative risk; CI, confidence interval.

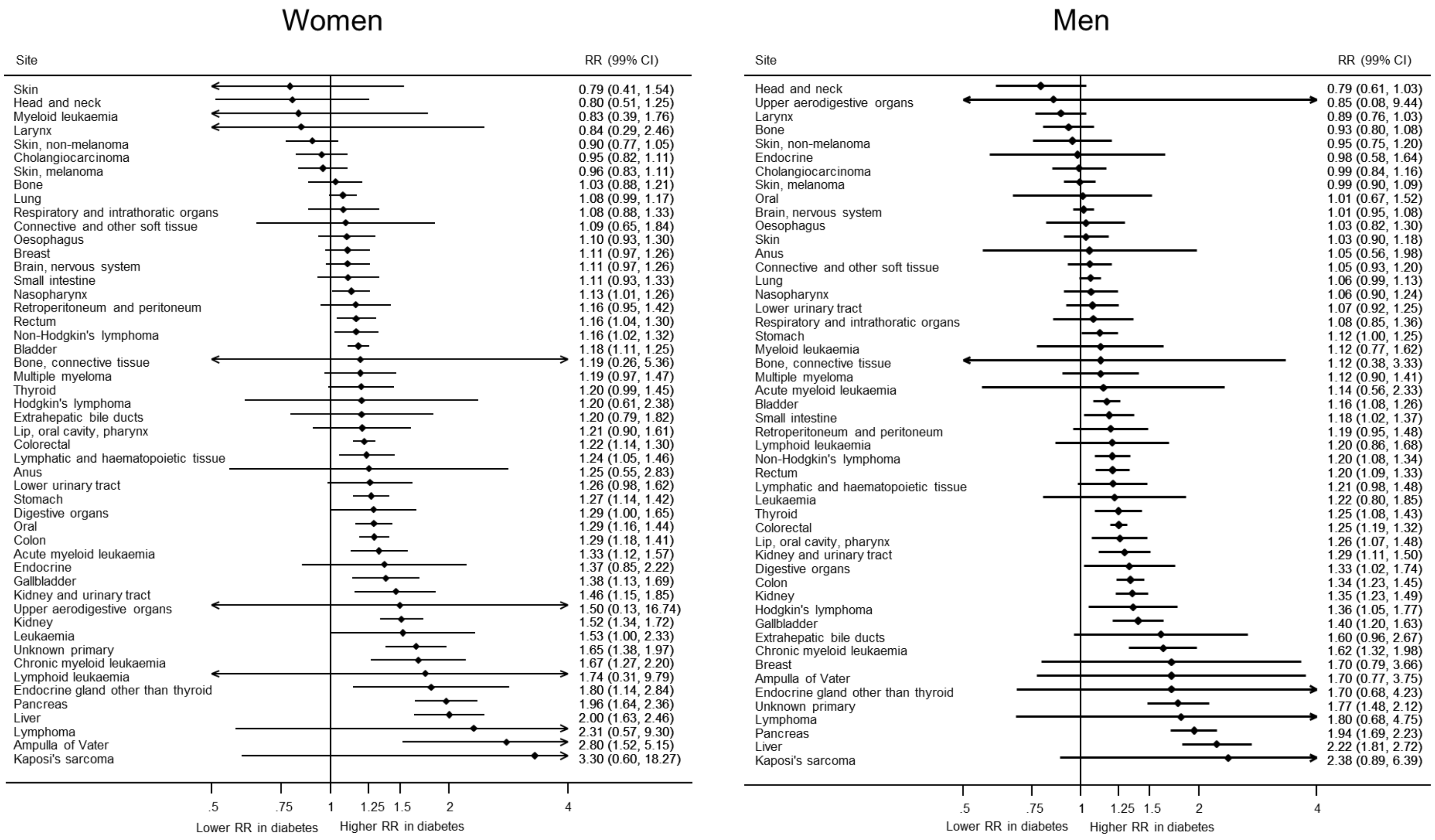

ESM Figure 8. Maximum available-adjusted pooled relative risk for cancer at each site, comparing individuals with diabetes to those without diabetes by sex.

Abbreviations; CI, confidence interval; RR, relative risk.

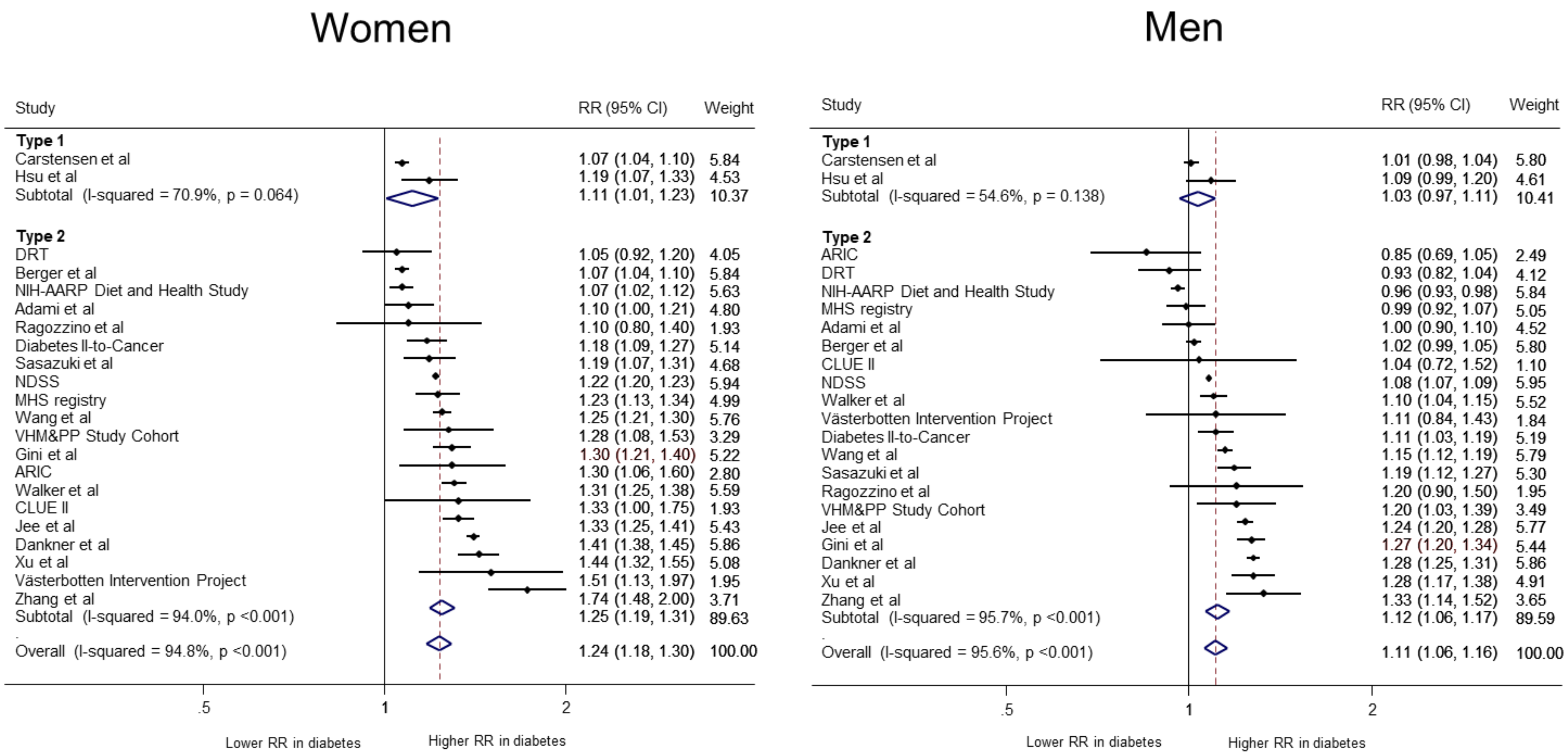

ESM Figure 9. Maximum available-adjusted relative risk for all-site cancer incidence, comparing individuals with diabetes to those without diabetes by sex.

Abbreviations; CI, confidence interval; RR, relative risk.

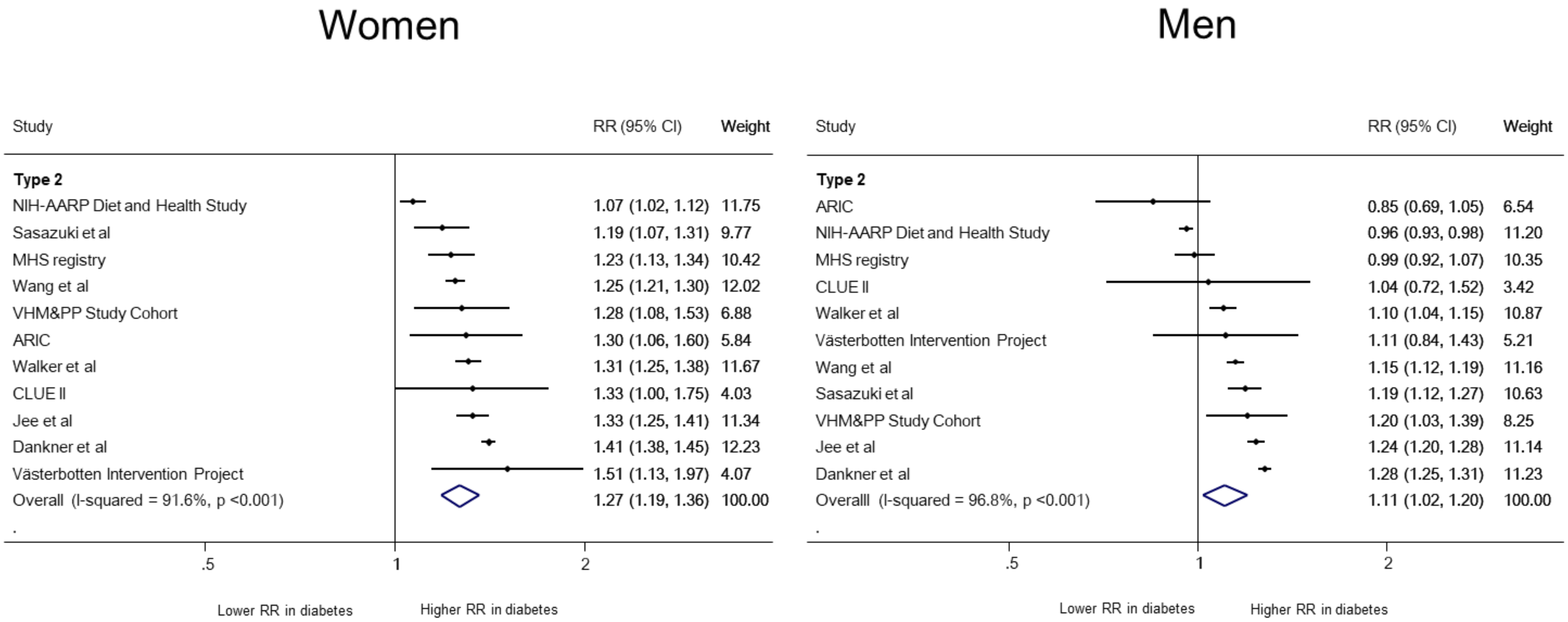

ESM Figure 10. Multiple-adjusted relative risk for all-site cancer incidence, comparing individuals with diabetes to those without diabetes by sex.

Studies that provided only age-adjusted results were excluded from the analysis.

Abbreviations; CI, confidence interval; RR, relative risk.

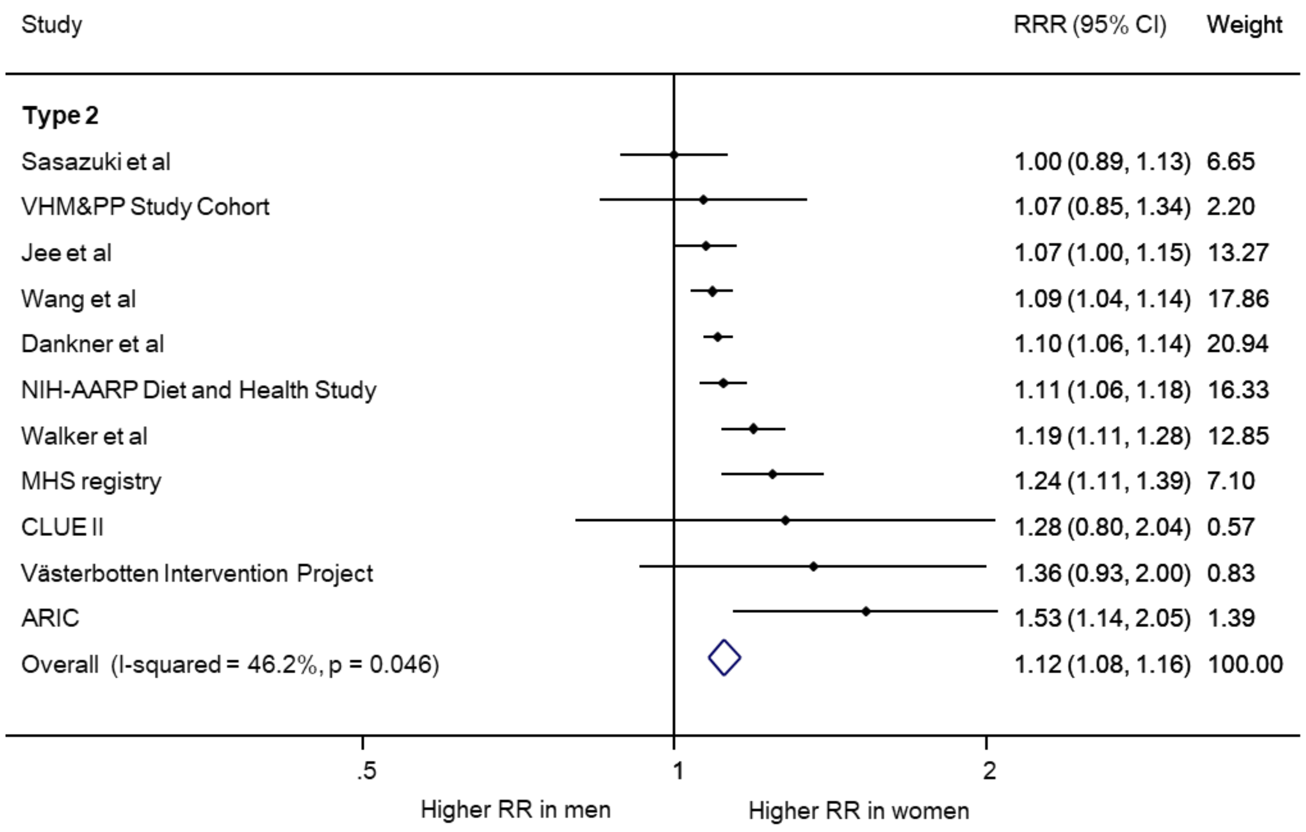

ESM Figure 11. Multiple-adjusted women-to-men ratio of relative risk for all-site cancer incidence, comparing individuals with diabetes to those without diabetes.

Studies that provided only age-adjusted results were excluded from the analysis.

Abbreviations; RR; relative risk; RRR, ratio of relative risk; CI, confidence interval.

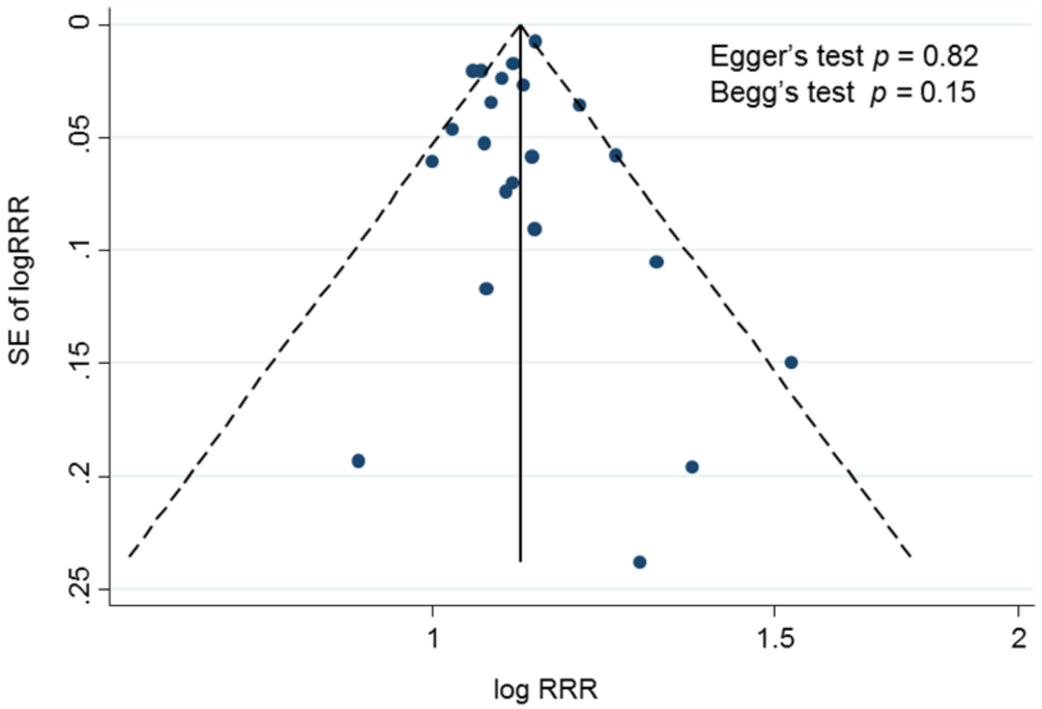

ESM Figure 12. Funnel plot with pseudo 95% confidence limits for the data in Supplementary Figure 5.

Abbreviations; RRR, ratio of relative risk; SE, standard error.

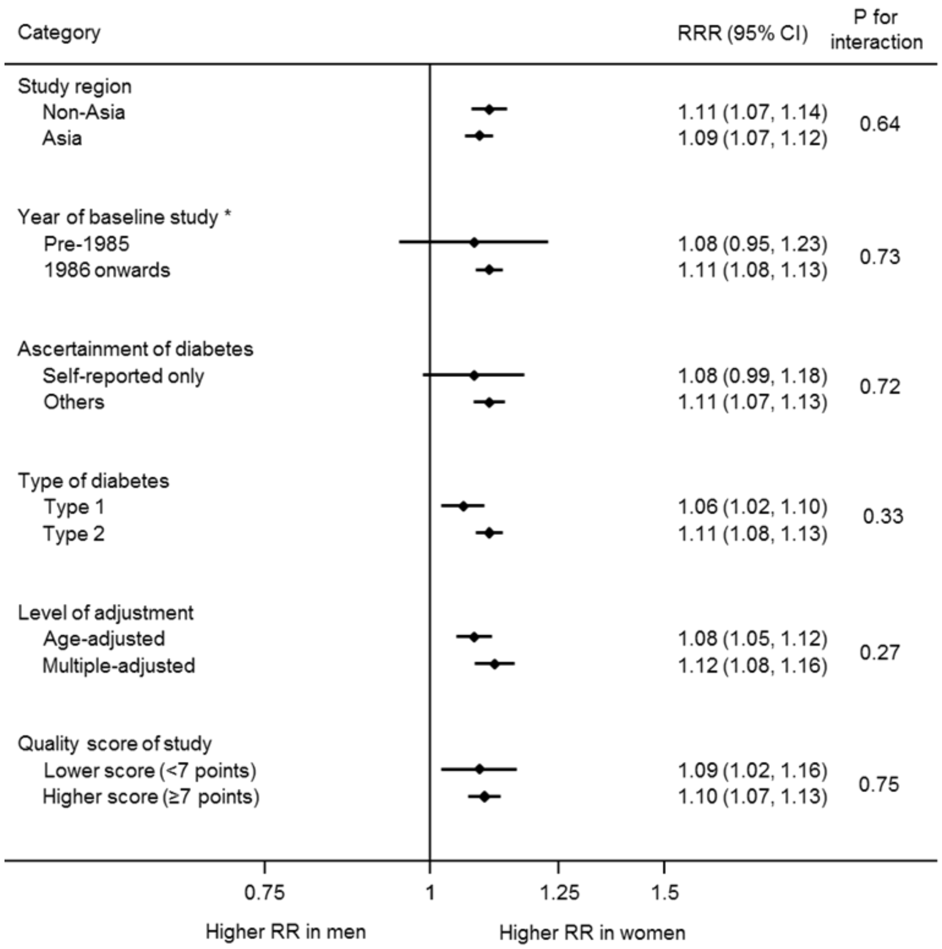

ESM Figure 13. Subgroup analyses of women-to-men ratio of relative risk for all-site cancer incidence, comparing individuals with diabetes to those without diabetes.

* One study were excluded because baseline year bridged over 1985.

Abbreviations; RR; relative risk; RRR, ratio of relative risk; CI, confidence interval.

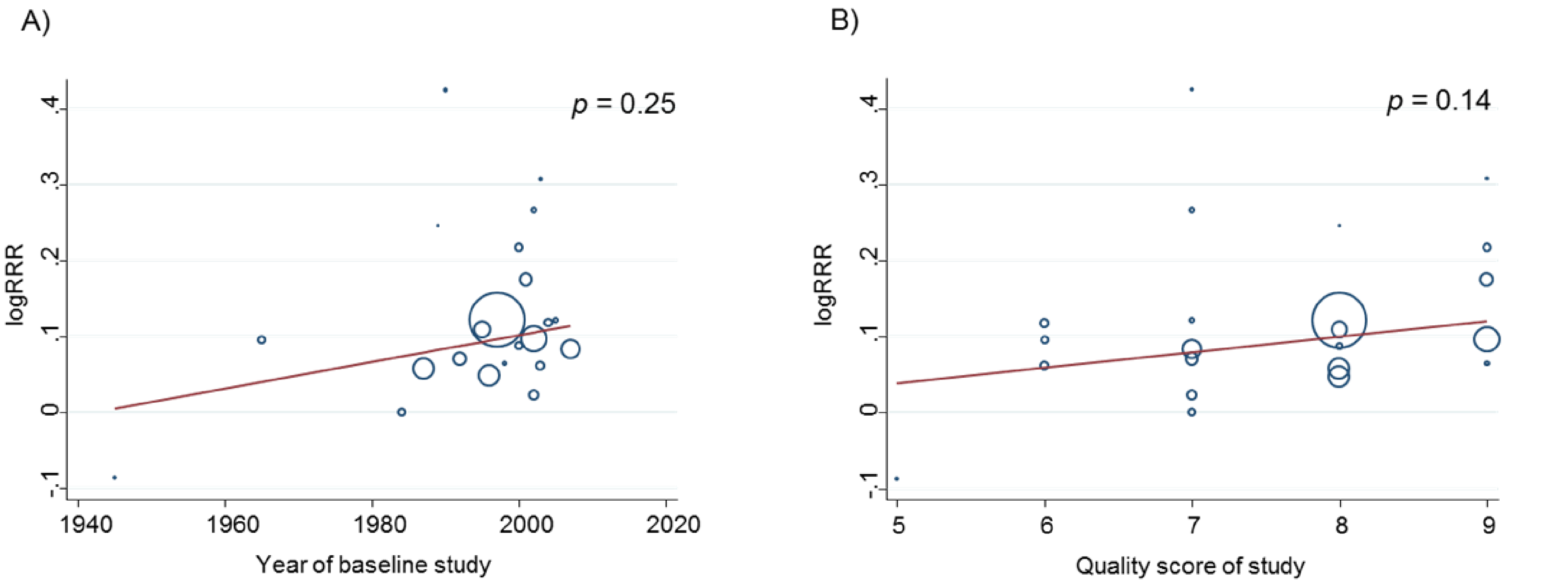

ESM Figure 14. Meta-regression of log (women-to-men ratio of relative risk for all-site cancer incidence) against A) year of baseline study and B) quality score of study (the Newcastle-Ottawa Scale) for the data in Supplementary Figure 5.

The circles for each study are drawn in proportion to the inverse variance.

Abbreviations; RRR, ratio of relative risk.

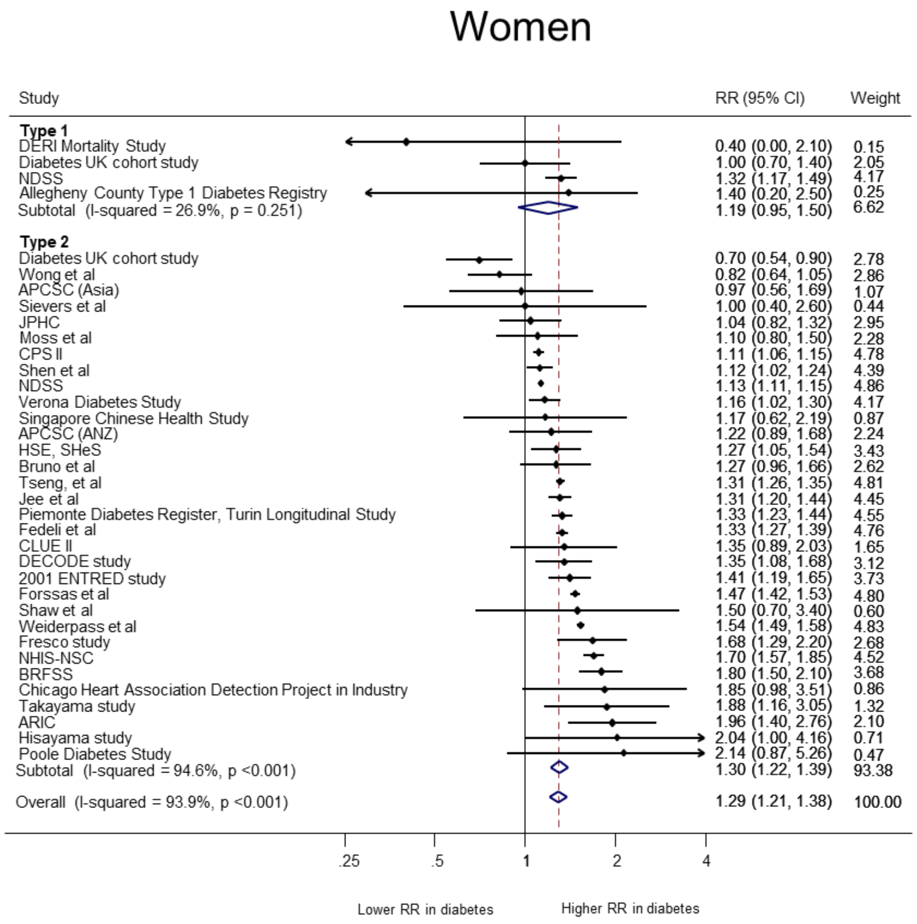

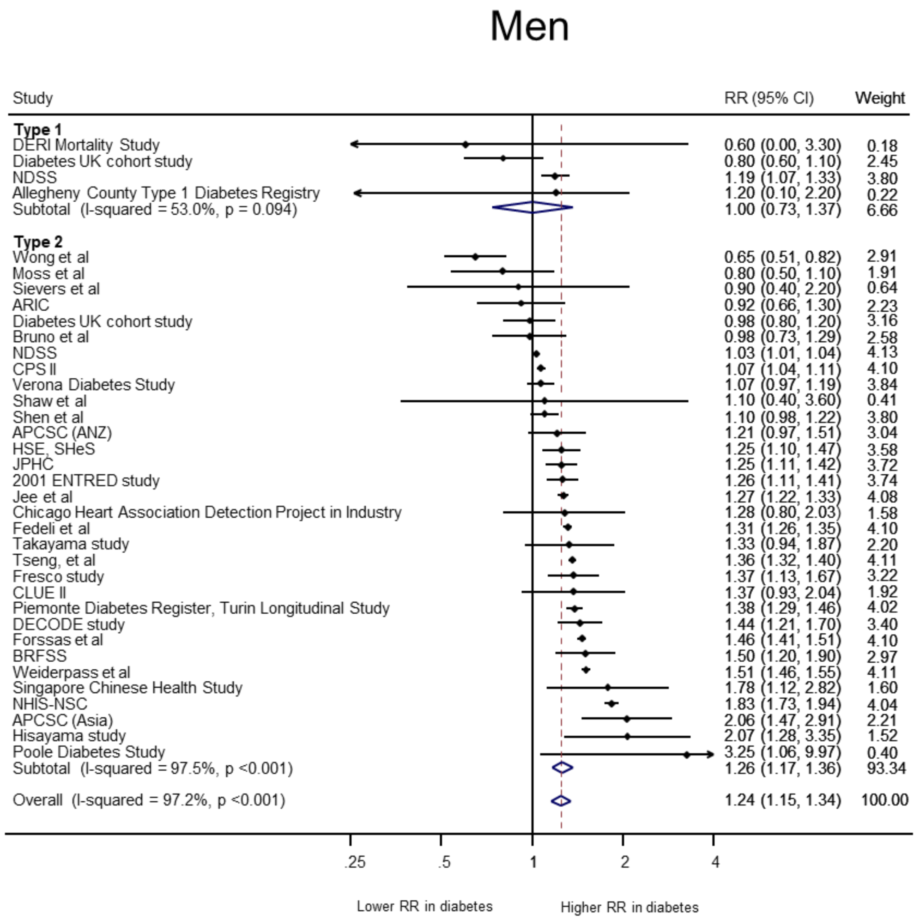

ESM Figure 15. Maximum available-adjusted relative risk for all-site cancer mortality, comparing individuals with diabetes to those without diabetes by sex.

Abbreviations; CI, confidence interval; RR, relative risk.

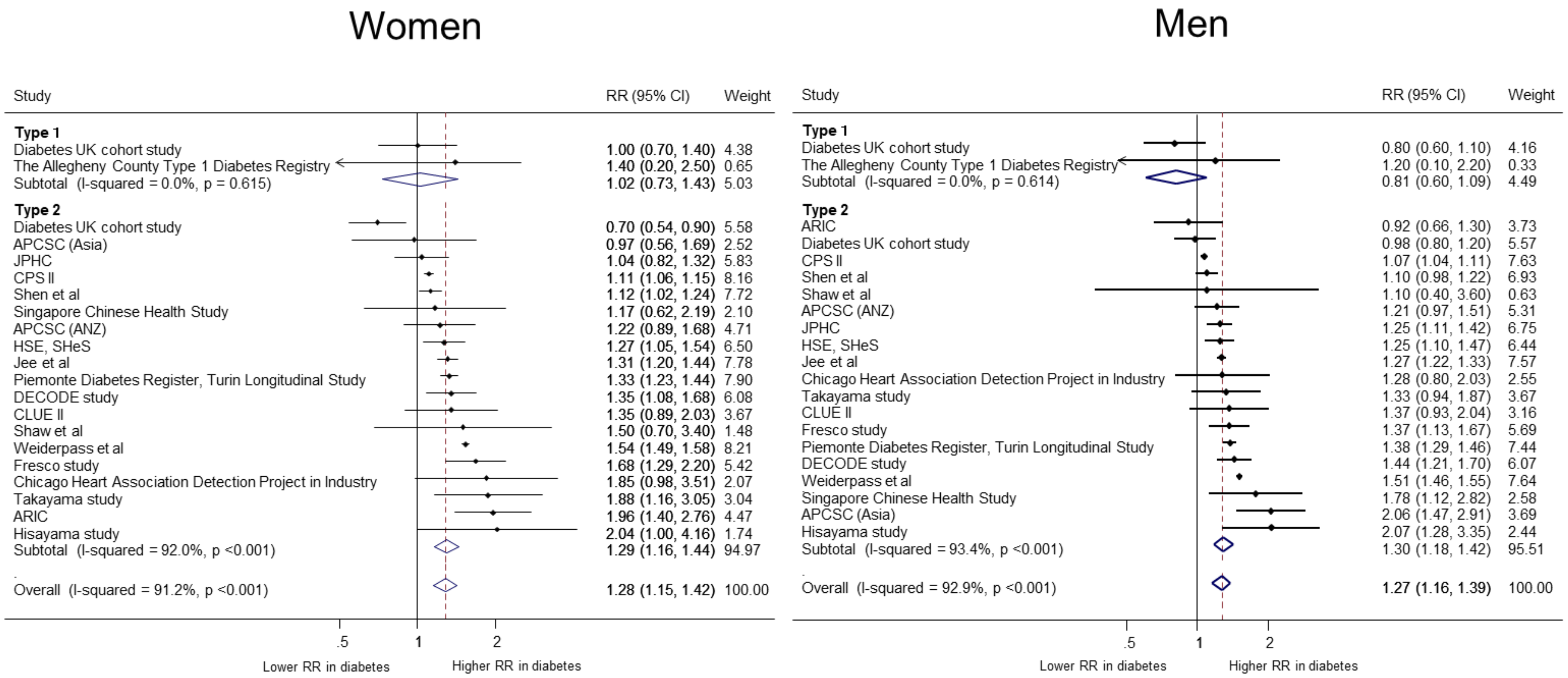

ESM Figure 16. Multiple-adjusted relative risk for all-site cancer mortality, comparing individuals with diabetes to those without diabetes by sex.

Studies that provided only age-adjusted results were excluded from the analysis.

Abbreviations; CI, confidence interval; RR, relative risk.

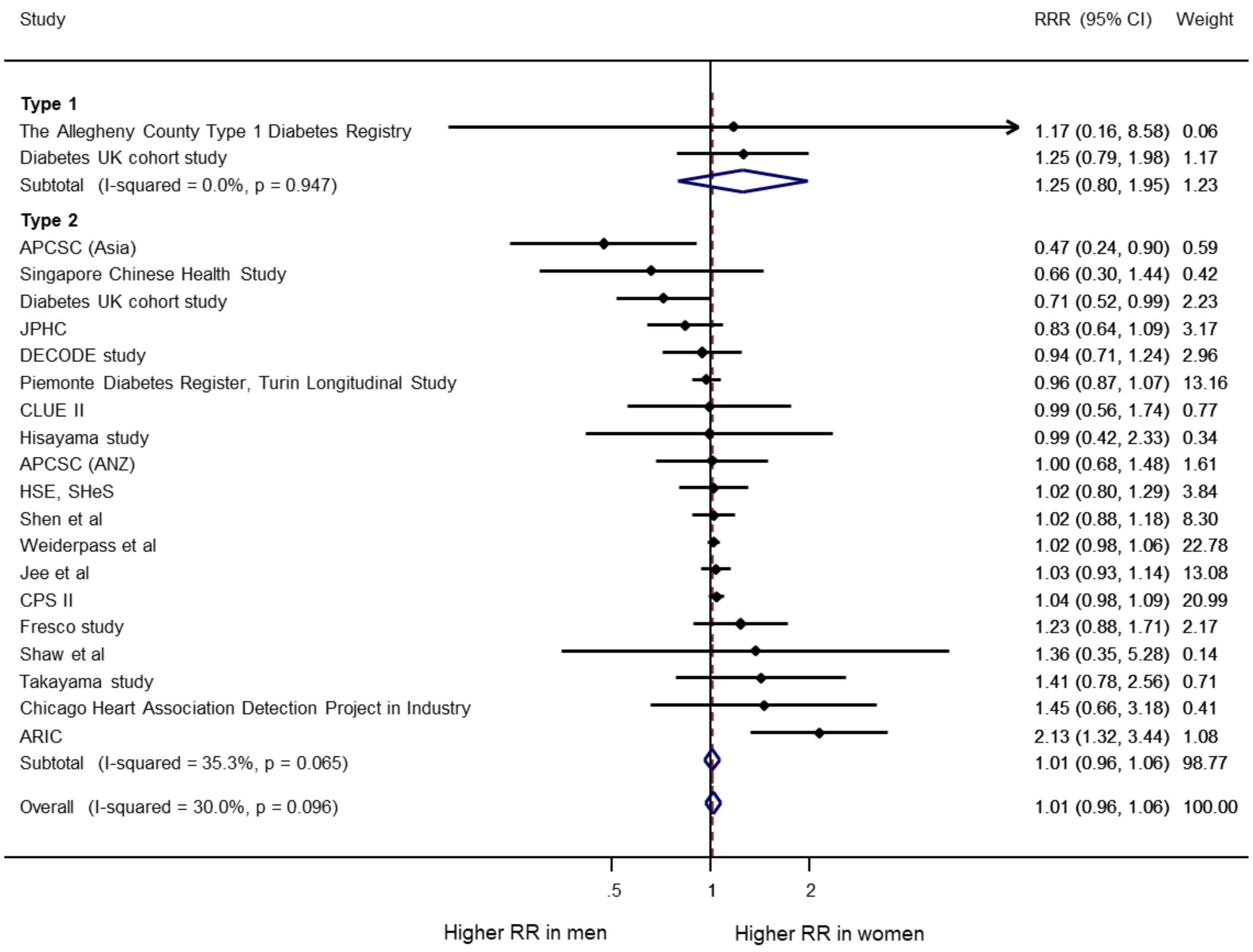

ESM Figure 17. Multiple-adjusted women-to-men ratio of relative risk for all-site cancer mortality, comparing individuals with diabetes to those without diabetes.

Studies that provided only age-adjusted results were excluded from the analysis.

Abbreviations; RR; relative risk; RRR, ratio of relative risk; CI, confidence interval.

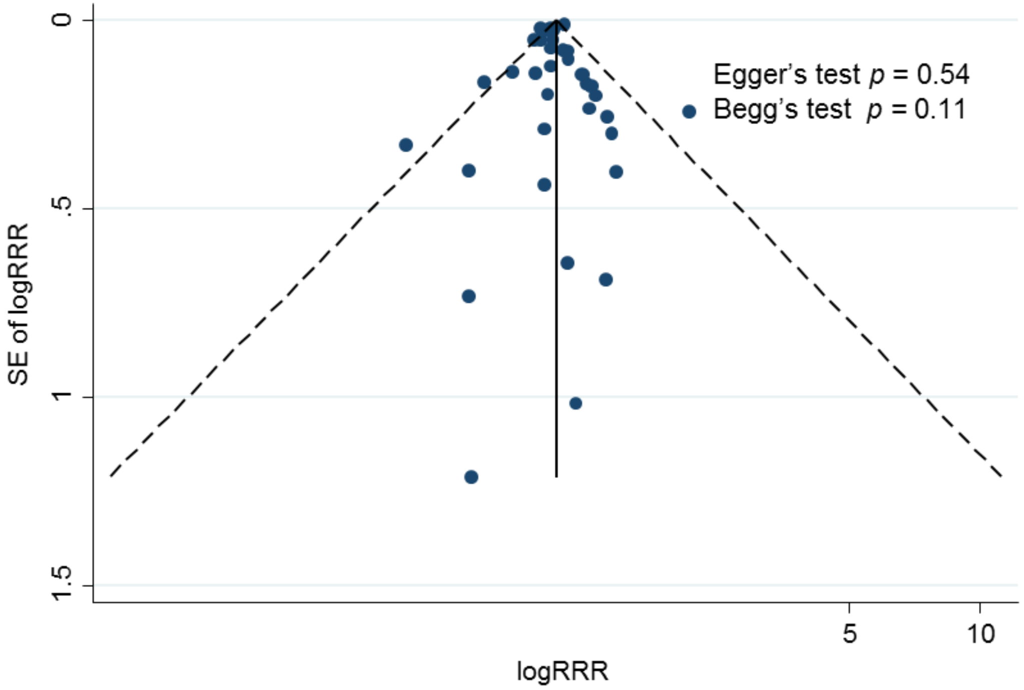

ESM Figure 18. Funnel plot with pseudo 95% confidence limits for the data in Supplementary Figure 6.

Abbreviations; RRR, ratio of relative risk; SE, standard error.

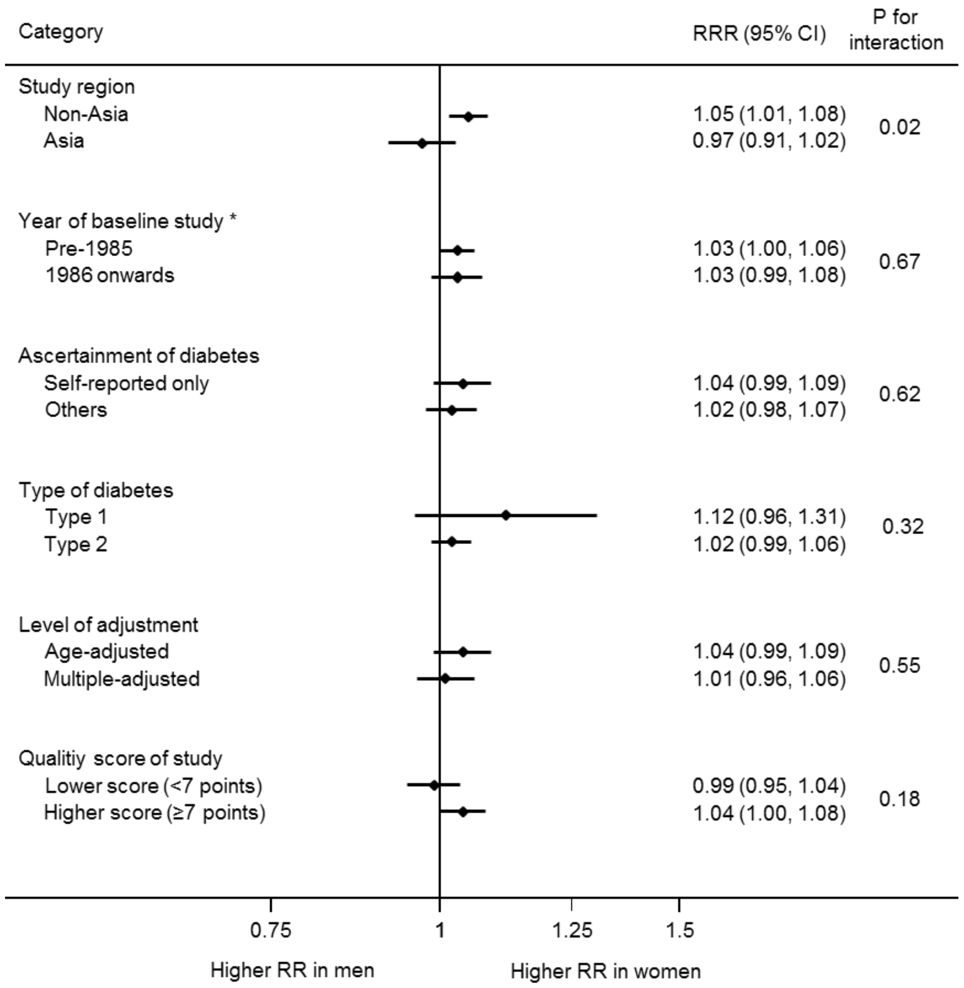

ESM Figure 19. Subgroup analyses of women-to-men ratio of relative risk for all-site cancer mortality, comparing individuals with diabetes to those without diabetes.

* 5 studies were excluded because baseline year bridged over 1985.

Abbreviations; RR; relative risk; RRR, ratio of relative risk; CI, confidence interval.

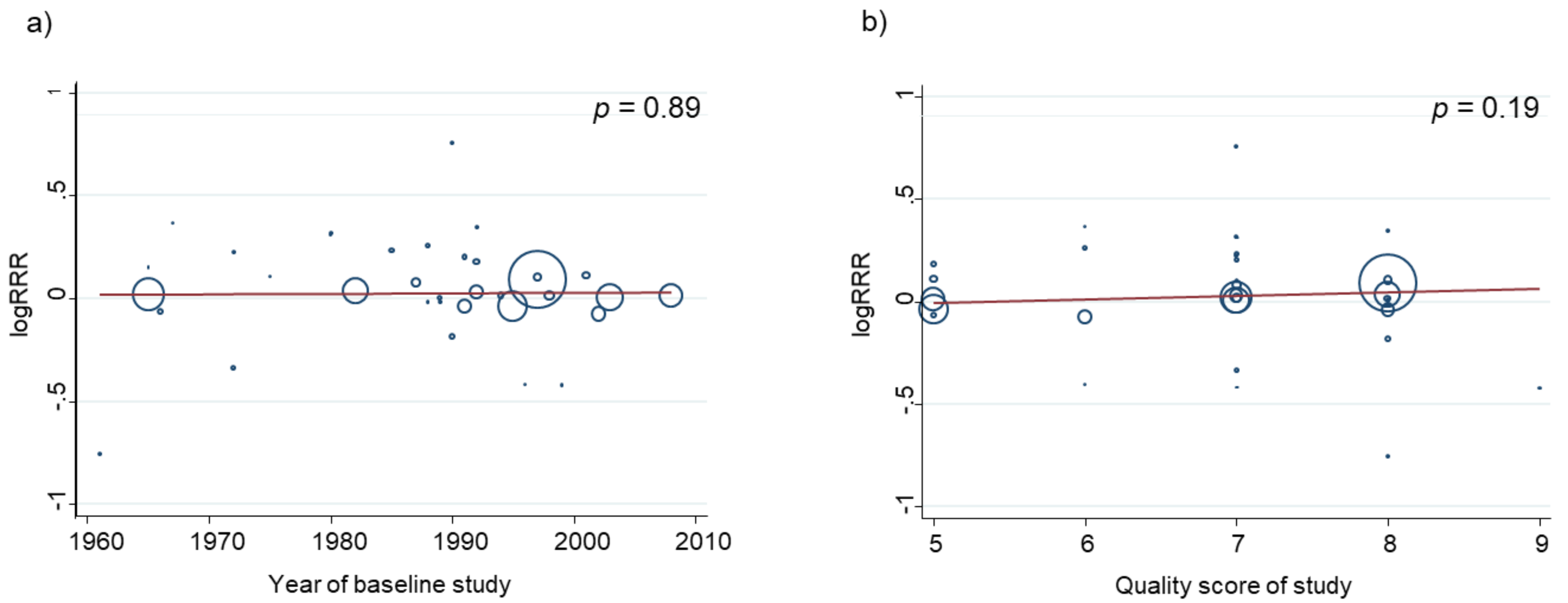

ESM Figure 20. Meta-regression of log (women-to-men ratio of relative risk for all-site cancer mortality) against A) year of baseline study and B) quality score of study (the Newcastle-Ottawa Scale) for the data in Supplementary Figure 6.

The circles for each study are drawn in proportion to the inverse variance.

Abbreviations; RRR, ratio of relative risk.

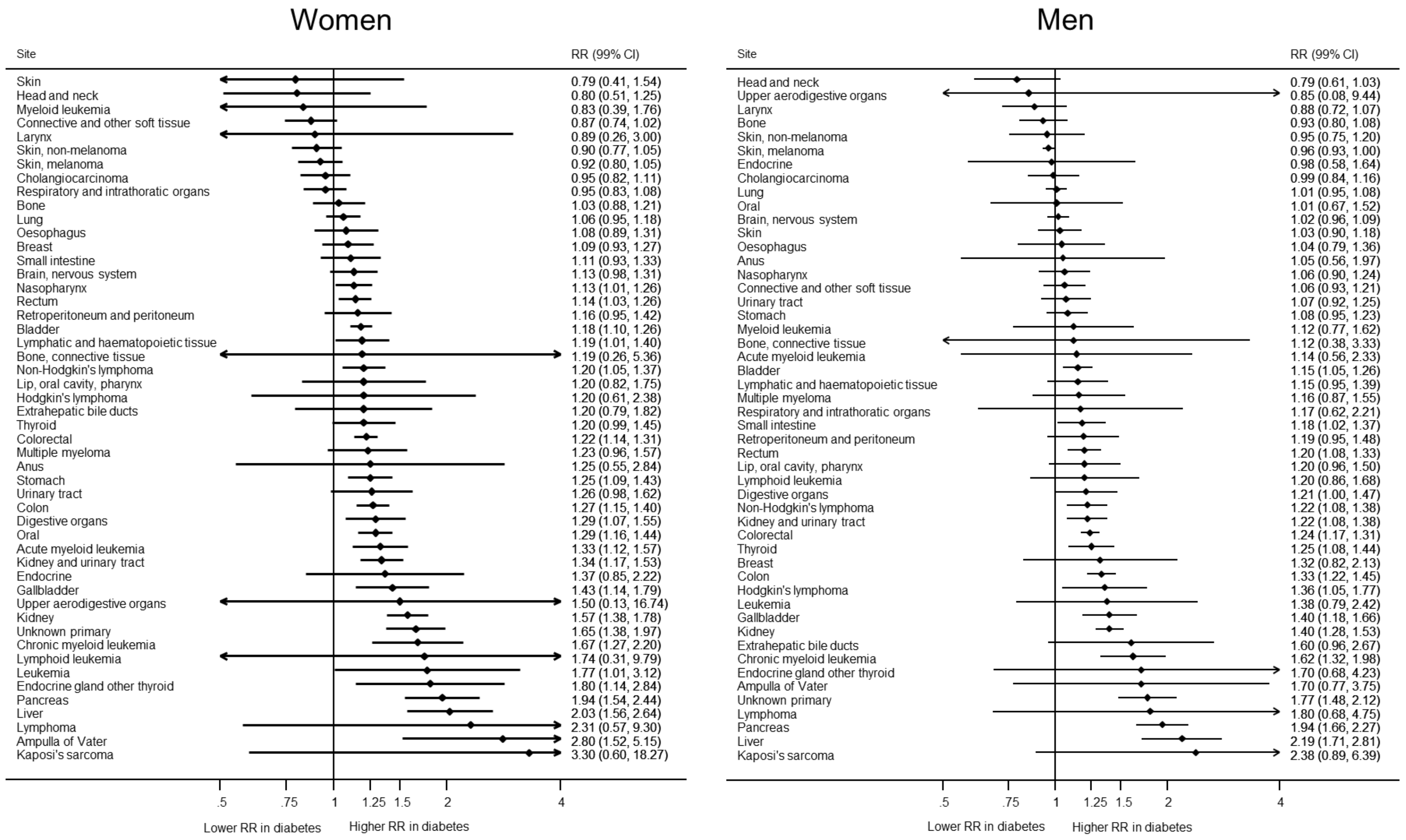

ESM Figure 21. Maximum available-adjusted pooled relative risk for incidence of cancer at each site, comparing individuals with diabetes to those without diabetes by sex.

Abbreviations; CI, confidence interval; RR, relative risk.

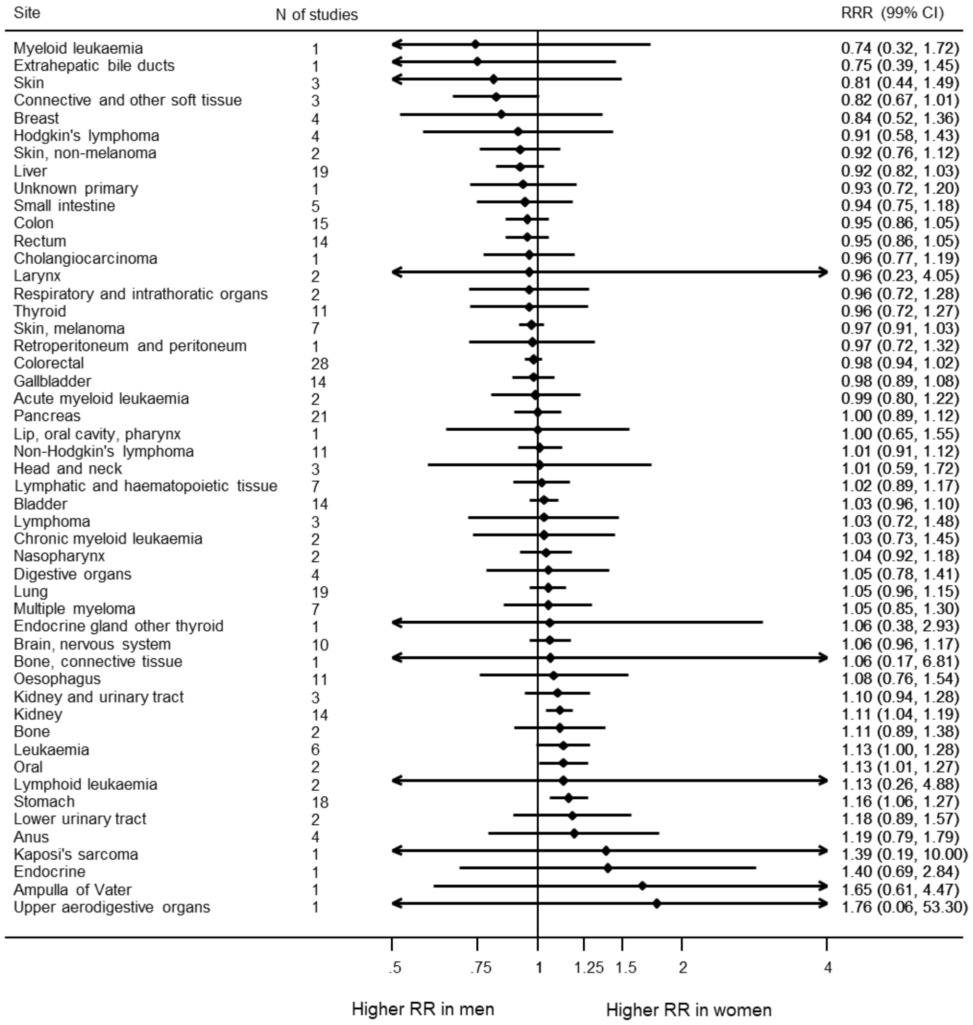

ESM Figure 22. Maximum available-adjusted pooled women-to-men ratio of relative risk for incidence of cancer at each site, comparing individuals with diabetes to those without diabetes.

Abbreviations; RR; relative risk; RRR, ratio of relative risk; CI, confidence interval.

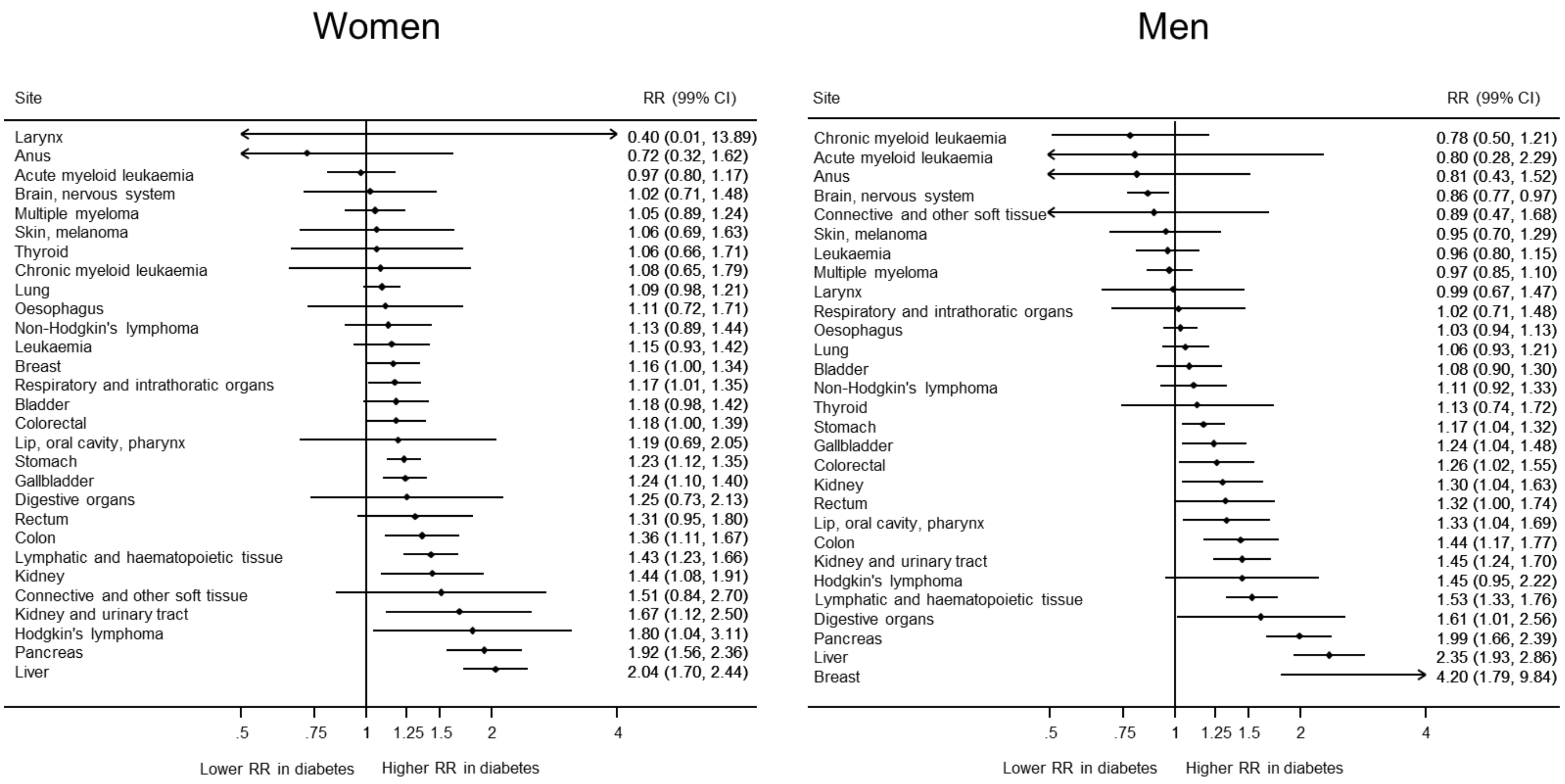

ESM Figure 23. Maximum available-adjusted pooled relative risk for mortality from cancer at each site, comparing individuals with diabetes to those without diabetes by sex.

Abbreviations; CI, confidence interval; RR, relative risk.

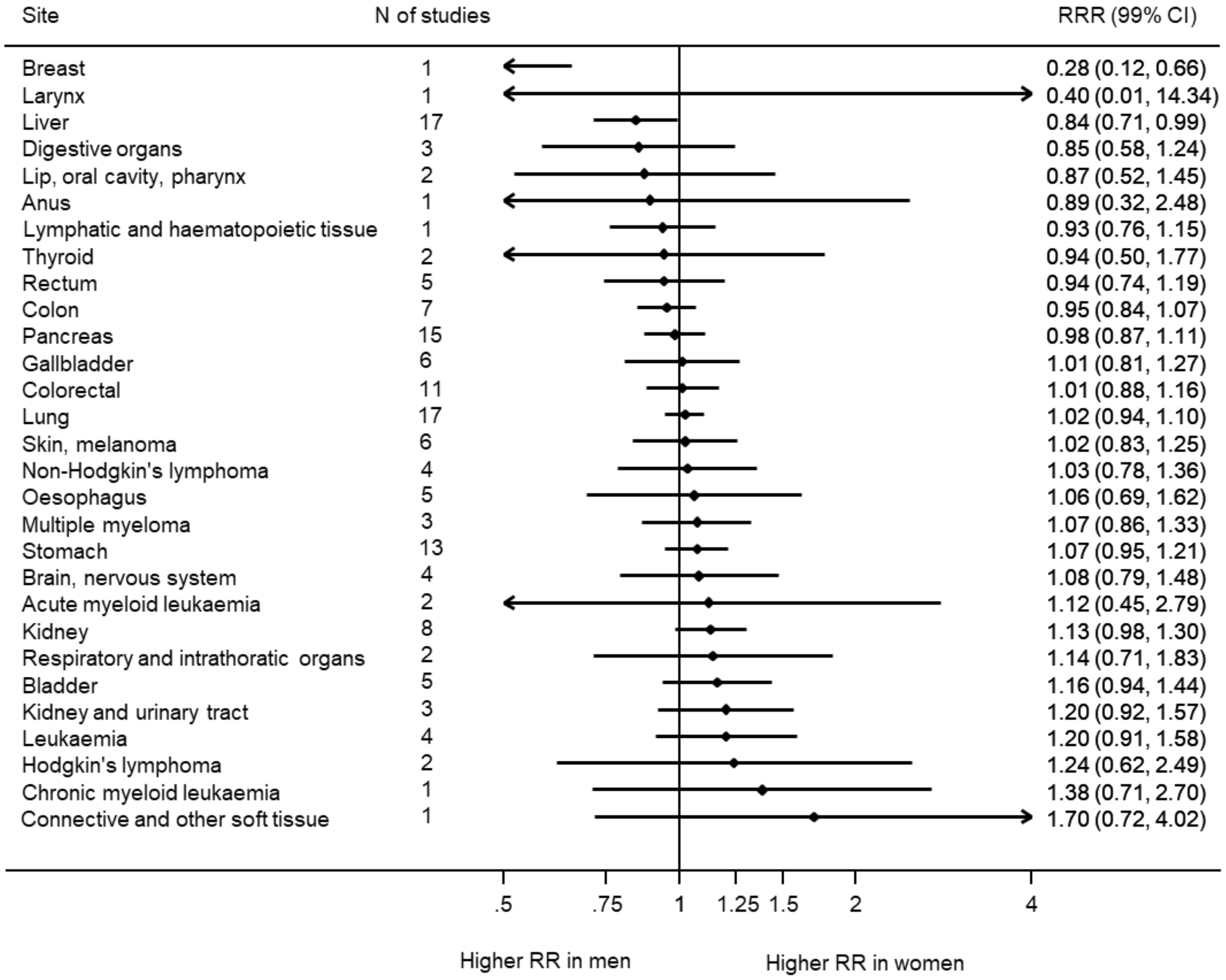

ESM Figure 24. Maximum available-adjusted pooled women-to-men ratio of relative risk for mortality from cancer at each site, comparing individuals with diabetes to those without diabetes.

Abbreviations; RR; relative risk; RRR, ratio of relative risk; CI, confidence interval.
